# Electrocatalytic Porphyrin/Phthalocyanine‐Based Organic Frameworks: Building Blocks, Coordination Microenvironments, Structure‐Performance Relationships

**DOI:** 10.1002/advs.202206239

**Published:** 2023-01-04

**Authors:** Ning Lv, Qian Li, Huang Zhu, Shengdong Mu, Xianglin Luo, Xiancheng Ren, Xikui Liu, Shuang Li, Chong Cheng, Tian Ma

**Affiliations:** ^1^ College of Polymer Science and Engineering State Key Laboratory of Polymer Materials Engineering Sichuan University Chengdu 610065 P. R. China; ^2^ Med‐X Center for Materials Sichuan University Chengdu 610041 P. R. China; ^3^ Department of Ultrasound West China Hospital Sichuan University Chengdu 610041 P. R. China

**Keywords:** coordination microenvironment, covalent organic frameworks, electrocatalytic applications, metal–organic frameworks, porphyrin and phthalocyanine

## Abstract

Metal‐porphyrins or metal‐phthalocyanines‐based organic frameworks (POFs), an emerging family of metal‐N‐C materials, have attracted widespread interest for application in electrocatalysis due to their unique metal‐N_4_ coordination structure, high conjugated *π*‐electron system, tunable components, and chemical stability. The key challenges of POFs as high‐performance electrocatalysts are the need for rational design for porphyrins/phthalocyanines building blocks and an in‐depth understanding of structure–activity relationships. Herein, the synthesis methods, the catalytic activity modulation principles, and the electrocatalytic behaviors of 2D/3D POFs are summarized. Notably, detailed pathways are given for modulating the intrinsic activity of the M‐N_4_ site by the microenvironments, including central metal ions, substituent groups, and heteroatom dopants. Meanwhile, the topology tuning and hybrid system, which affect the conjugation network or conductivity of POFs, are also considered. Furthermore, the representative electrocatalytic applications of structured POFs in efficient and environmental‐friendly energy conversion areas, such as carbon dioxide reduction reaction, oxygen reduction reaction, and water splitting are briefly discussed. Overall, this comprehensive review focusing on the frontier will provide multidisciplinary and multi‐perspective guidance for the subsequent experimental and theoretical progress of POFs and reveal their key challenges and application prospects in future electrocatalytic energy conversion systems.

## Graphical abstract

Recent advancements toward the electrocatalytic porphyrins/phthalocyanines‐based organic frameworks (POFs) as the family of metal‐N‐C materials are systematically reviewed. The state‐of‐the‐art synthetic methods, the catalytic activity modulation principles, and the corresponding electrocatalytic behaviors of 2D/3D POFs have been thoroughly discussed. Particularly, detailed pathways are given for tuning their precise chemical and electronic structure, including coordination microenvironment, topology, and hybrid system.

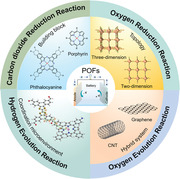

## 1 Introduction

With the fast societal development and industrial consumption, the supply of sustainable energy and environmental protection are facing severe challenges.^[^ ^1^, ^2^, ^3^, ^4^ ^]^ The emergence and development of emerging energy technologies provide possible technical solutions to overcome this problem.^[^ ^5^, ^6^, ^7^ ^]^ Among them, electrocatalytic systems, including carbon dioxide reduction reaction (CO_2_RR, carbon capture, and storage),^[^ ^8^ ^]^ oxygen reduction reaction (ORR, the conversion of chemical energy to electrical energy),^[^ ^9^, ^10^ ^]^ and water‐splitting (consists of hydrogen and oxygen production, the conversion of electrical energy to hydrogen energy),^[^ ^11^, ^12^ ^]^ have attracted much attention of researchers due to their high efficiency and good adaptability to future energy systems. Therefore, it is urgent to design high‐performance, stable, and novel electrocatalysts that meet the needs of a variety of applications.^[^ ^13^, ^14^, ^15^ ^]^ Until now, the most popular electrocatalysts are the costly noble metal‐derived catalysts. However, the use of expensive, unsatisfactory stability, and scarce precious metal‐based electrocatalysts limits their wide applications.^[^ ^16^, ^17^, ^18^ ^]^

**Scheme 1:**
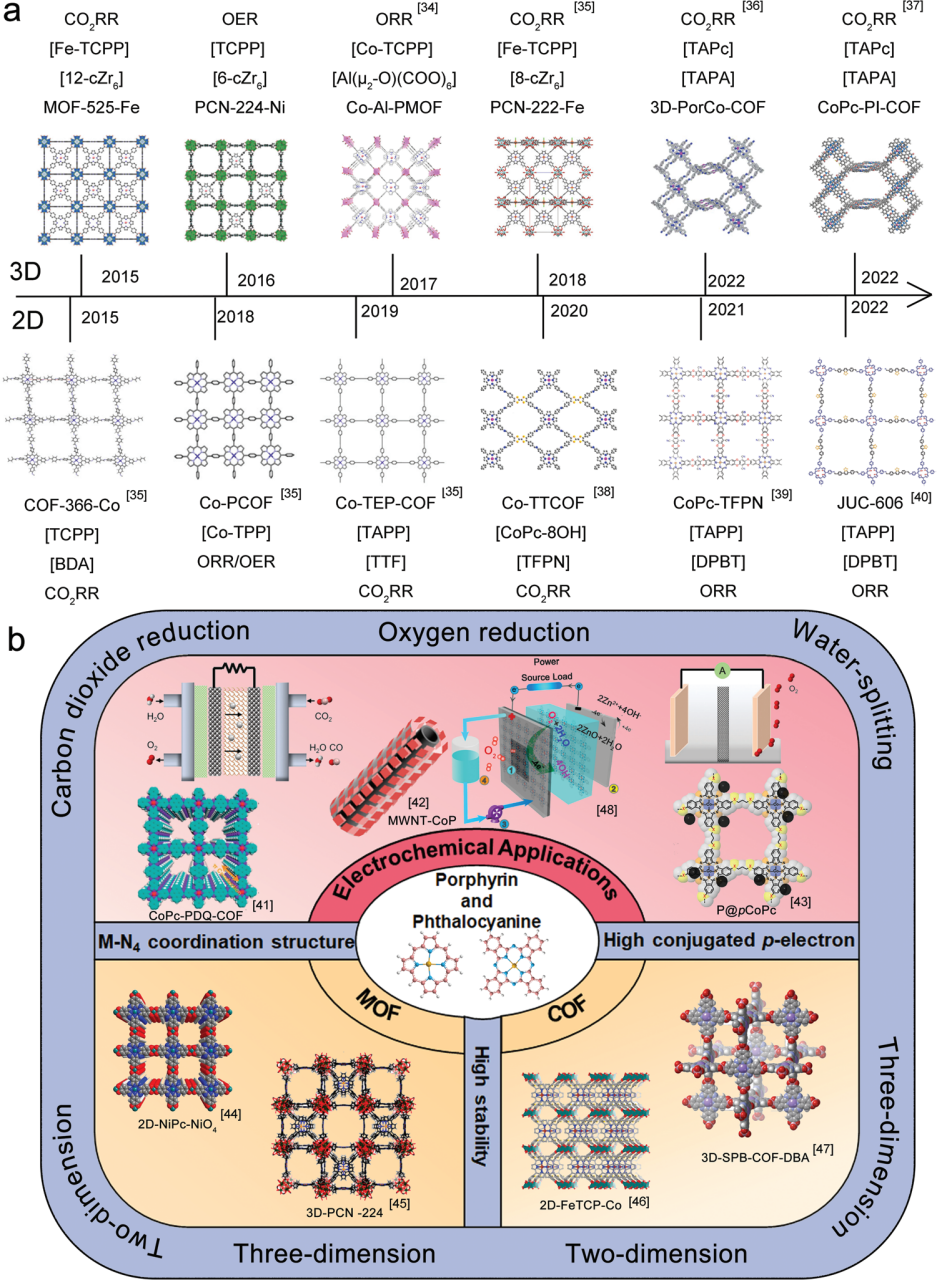
a) The development of 2D and 3D POFs with electrocatalytic activities in recent years. Reproduced with permission.^[^ ^34^ ^]^ Copyright 2020, Elsevier. Reproduced with permission.^[^ ^35^ ^]^ Copyright 2020, The Royal Society of Chemistry. Reproduced with permission.^[^ ^36^ ^]^ Copyright 2022, The Royal Society of Chemistry. Reproduced with permission.^[^ ^37^ ^]^ Copyright 2021, Wiley‐VCH. Reproduced with permission.^[^ ^38^ ^]^ Copyright 2020, Springer Nature. Reproduced with permission.^[^ ^39^ ^]^ Copyright 2020, Wiley‐VCH. Reproduced with permission.^[^ ^40^ ^]^ Copyright 2021, Springer Nature. b) The overall porphyrins/phthalocyanines‐based organic frameworks and their electrochemical applications. Reproduced with permission.^[^ ^41^ ^]^ Copyright 2020, Wiley‐VCH. Reproduced with permission.^[^ ^42^ ^]^ Copyright 2014, American Chemical Society. Reproduced with permission.^[^ ^43^ ^]^ Copyright 2021, Wiley‐VCH. Reproduced with permission.^[^ ^44^ ^]^ Copyright 2021, Wiley‐VCH. Reproduced with permission.^[^ ^45^ ^]^ Copyright 2016, American Chemical Society. Reproduced with permission.^[^ ^46^ ^]^ Copyright 2021, Wiley‐VCH. Reproduced with permission.^[^ ^47^ ^]^ Copyright 2021, American Chemical Society. Reproduced with permission.^[^ ^48^ ^]^ Copyright 2019, American Chemical Society.

To improve the catalytic efficiency and reduce the high cost of materials, various advanced electrocatalysts for energy conversion technology have been developed.^[^ ^11^ ^]^ In recent years, electrocatalysts based on the transitional metal‐N‐C (M‐N‐C) structures with outstanding catalytic activity have attracted extensive attention due to their unique electronic structure combined with reaction intermediates, abundant substituted metal elements, maximum atom utilization efficiency, and excellent stability.^[^ ^18^, ^19^, ^20^, ^21^, ^22^, ^23^ ^]^ Existing methods for preparing M‐N‐C catalysts usually include mixing sources of nitrogen, carbon, and transition metal, and then heat treatment.^[^ ^13^ ^]^ In these processes, the transition metal precursors and nitrogen sources are usually dispersed randomly on the carbon support or mixed with the carbon support, which makes it difficult to obtain the ordered structure of the electrocatalysts.^[^ ^21^, ^24^, ^25^, ^26^ ^]^ This will ineluctably affect the electrocatalytic reaction kinetics and lead to insufficient electrocatalytic activity. Given these limitations, the development of electrocatalysts with well‐defined M‐N‐C structures is a challenging but crucial task. As a kind of M‐N‐C based materials with precise M‐N_4_ catalytic center and a larger *π*‐conjugated structure, transition metal coordinated‐ porphyrins (Por), phthalocyanines (Pc), and their derivatives have been extensively studied in the field of electrocatalysis.^[^ ^17^, ^27^, ^28^, ^29^, ^30^ ^]^ Especially, Por/Pc‐based metal–organic frameworks (MOFs) and covalent organic frameworks (COFs), which were referred as Por/Pc‐based organic frameworks (POFs) in this review (**Scheme** **1**), have currently become the focus of research in this field, due to their unique characteristics of periodic network structure, large specific surface area, and adjustable mesoporous size. To be specific, MOF, as a new crystalline porous material, is composed of organic connectors and metal ions/clusters.^[^ ^31^ ^]^ Compared with MOFs connected by coordination bonds, COFs are synthesized by the building block and linker through organic coupling reactions, which form strong covalent bonds between the basic units, exhibiting higher chemical and thermodynamic stability.^[^ ^32^, ^33^ ^]^ In summary, the M‐Por/Pc with precise M‐N_4_ catalytic center and tunable structure together constitute a series of electrocatalysts with uniform distribution of active metals, which are beneficial for exploring the structure‐performance relationship of M‐N‐C.

Based on this research, several reviews have focused on the design and development of POFs for energy storage and conversion. However, most of them mainly review the chemical structure, synthesis methods, or physicochemical properties of POFs; it is urgently needed to summarize the POFs‐based electrocatalysts with a combination of the synthetic procedure and the catalytic modulation strategy, particularly to state the structure‐performance relationship. Herein, we summarize the synthesis methods, the catalytic activity modulation principles, and the electrocatalytic behaviors of 2D/3D POFs. Notably, we give detailed pathways for modulating the intrinsic activity of the M‐N_4_ site by the microenvironments, including central metal ions, substituent groups, and heteroatom dopants. Meanwhile, the topology tuning and hybrid system, which affect the conjugation network or conductivity of POFs, are also considered. Furthermore, we briefly discuss the representative electrocatalytic applications of structured POFs in efficient and environmental‐friendly energy conversion areas and the latest research breakthroughs, such as CO_2_RR, ORR, and water splitting. Overall, this comprehensive review focusing on the frontier will provide multidisciplinary and multi‐perspective guidance for the subsequent experimental and theoretical progress of POFs and reveal their key challenges and application prospects in future electrocatalytic energy conversion systems.

## 2 Synthesis of Structured Porphyrins/Phthalocyanines‐Based Organic Frameworks

**Figure 1:**
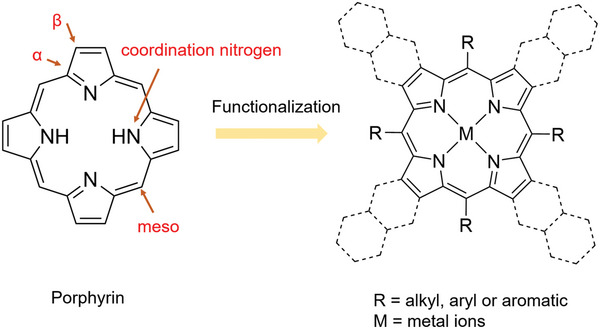
Basic porphyrin structure and expected modified routes. Reproduced with permission.^[^ ^52^ ^]^ Copyright 2020, Elsevier.

Hemoglobin, which is composed of the Fe‐N_4_ coordination structure of the porphyrin, exists in various metalloenzymes and proteins of organisms and plays a key role in wide biological processes.^[^ ^49^, ^50^, ^51^ ^]^ Inspired by such natural enzymes, many compounds with N_4_‐conjugated macrocycles can combine with metal ions to form M‐N_4_ structures to simulate biocatalysts systems.^[^ ^27^, ^28^ ^]^ At present, the research of M‐N_4_ structure in conjugated macrocycles mainly focuses on Por, Pc, and their derivatives. As shown in **Figure** **1**, Por with 18 conjugated electronic systems is connected by the pyrrole rings and methine bridges at *α*‐ position. In addition, *β*‐ and meso‐ positions of Por are the other essential sites where functionalization can be carried out.^[^ ^52^ ^]^ As another one of the most studied classes of conjugated macrocycles, Pc is usually chosen as the planar bridged unit because of its redox activity and good electron transfer ability on the strength of the large delocalized *π* system. Notably, the cavity of in Por/Pc macrocyclic structure can fix more than seventy elements so that the coordinated metal atoms have the same local coordination environment.^[^ ^17^, ^29^, ^30^ ^]^ As a result, the unique advantages of POFs (including Por/Pc‐MOFs and Por/Pc‐COFs), combining the rigid and stable coordination environments as well as easily modified structure with different functional groups at the meso‐ and *β*‐positions, have been extensively used as electrocatalysts for electrocatalysis, including CO_2_RR, ORR, HER, and OER.

It is essential to engineering POFs‐based materials with many accessible active sites, good crystallinity, and high chemical stability to achieve economic applications in the electrocatalytic field, which can be realized by choosing suitable Por/Pc building blocks, metal nodes, and linkers. In the next part, we will thoroughly summarize the common strategies for constructing different kinds of POFs‐based catalysts.

### 2.1 Synthesis of Porphyrins/Phthalocyanines‐Metal–Organic Framework

**Scheme 2:**
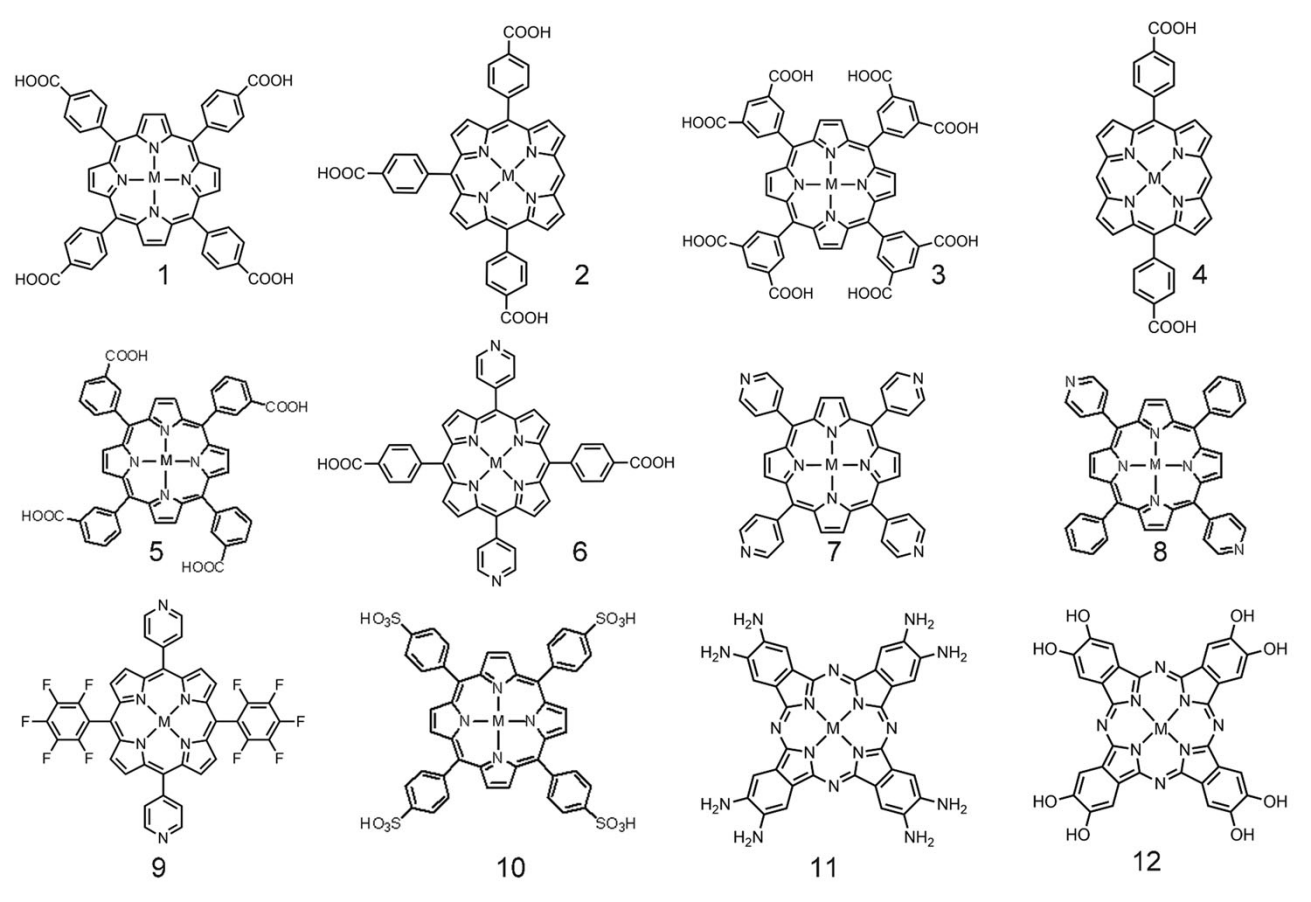
The structures of M‐Por and M‐Pc (M = Fe, Co, Ni, Cu, and Zn) for the synthesis of MOFs. Tetrakis(4‐carboxyphenyl)porphyrin (TCPP, 1), 5,10,15‐tri(4‐carboxylphenyl)porphyrin (2), 5,10,15,20‐tetrakis(8‐carboxylphenyl)porphyrin (TBCPPP, 3), 5,15‐bis(3,5‐dicarboxyphenyl)porphine (DCPP, 4), 3,3′,3″,3‴‐(21H,23H‐porphine‐5,10,15,20‐tetrayl)tetrakis‐benzoic acid (TMCPP, 5), 5,15‐di(4‐carboxylphenyl)‐10,20‐di(4‐pyridyl)porphyrin) (DCDPP, 6), 5,10,15,20‐tetra(4‐pyridyl)‐21H,23H‐porphine (TPyP, 7), 5,15‐di(4‐pyridyl)‐10,20‐dipenylporphyine (DPyP, 8), 5,15‐dipyridyl‐10,20‐bis(pentafluorophenyl)porphyrin (DPBPP, 9), Tetrakis(4‐sulfophenyl)porphine (TPPS_4_, 10), 2,3,9,10,16,17,23,24‐octa(amino)phthalocyanine (8OH‐Pc, 11), 2,3,9,10,16,17,23,24‐octa(hydroxyl) phthalocyanine (8NH_2_‐Pc, 12).

Por/Pc as macrocyclic molecules occupies an important position in the synthesis and application of MOFs. The rigid macrocycles of M‐Por and M‐Pc can be easily modified by the series of organic groups (carboxyl, pyridine, fluorine‐substituted, sulfonic group, amido, hydroxyl, and so on) to form diverse Por/Pc derivatives with different symmetries and sizes to afford abundant coordinated sites, making them ideal building blocks for constructing MOFs (**Scheme** **2**).^[^ ^53^, ^54^ ^]^ Among the above‐mentioned modified Por/Pc, carboxy‐ and pyridine‐modified Por are the most widely used building blocks because of the simple synthetic process. Differently, there are few reports about the Pc‐MOFs synthesized through the modified Pc building blocks, but significant progress has been gotten ahead very recently.^[^ ^34^, ^55^ ^]^ Generally, Por/Pc‐based MOFs can be separated into two categories by the different combination way between Por/Pc small molecules and MOFs. On the one hand, Por/Pc can act as guest molecules into MOFs through surface adsorption, grafting, or encapsulation in MOF pores to build MOFs loaded Por/Pc. On the other hand, Por/Pc acting as organic linkers coordinate with metal ions/clusters to construct the conjugated Por/Pc‐MOFs, which exhibit high conductivity and catalytic activity.^[^ ^56^ ^]^ Here, we mainly focus on synthesizing Por/Pc‐MOFs, which will be discussed as 2D layered structures or 3D networks.

#### 2.1.1 Synthesis of 2D Porphyrins/Phthalocyanines‐Metal–Organic Framework

**Figure 2:**
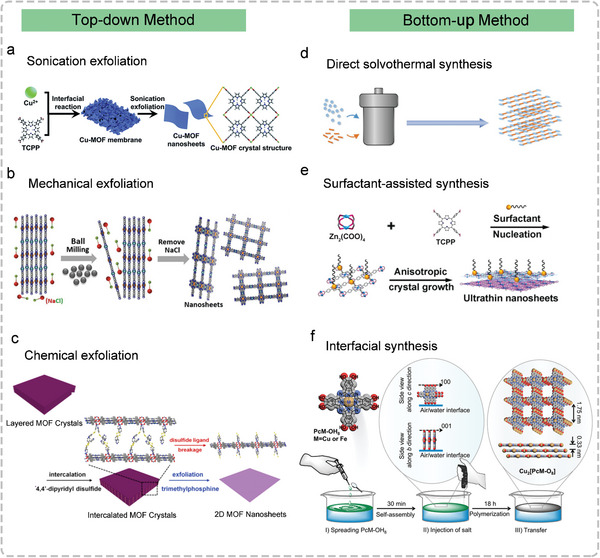
The top‐down and bottom‐up methods for the synthesis of 2D Por/Pc‐MOFs. a) The sonication exfoliation method for developing Cu‐MOF nanosheets. Reproduced with permission.^[^ ^62^ ^]^ Copyright 2019, The Royal Society of Chemistry. b) The mechanical exfoliation method for developing Ni_2_[CuPc(NH)_8_] 2D c‐MOF. Reproduced with permission.^[^ ^64^ ^]^ Copyright 2020, Wiley‐VCH. c) The chemical exfoliation method for developing 2D Por‐MOF nanosheets. Reproduced with permission.^[^ ^65^ ^]^ Copyright 2017, American Chemical Society. d) The solvothermal approach for 2D Por/Pc‐MOFs. Reproduced with permission.^[^ ^66^ ^]^ Copyright 2015, Wiley‐VCH. e) The surfactant‐assisted synthesis approach for ultrathin nanosheets. Reproduced with permission.^[^ ^67^ ^]^ Copyright 2020, Wiley‐VCH. f) The interfacial synthesis for 2D Cu_2_[PcM‐O_2_]. Reproduced with permission.^[^ ^68^ ^]^ Copyright 2021, American Chemical Society.

In recent years, 2D Por/Pc‐MOF nanosheets not only display novel physical and chemical properties but also possess highly accessible active sites, which have attracted more and more attention in a series of different applications.^[^ ^57^ ^]^ For instance, first, by altering the Por/Pc ligands or metal nodes of the MOF nanosheets, they owe tunable structures and properties, which provide advantages for achieving unique applications. Second, because of their high specific surface area and ultrathin thickness, 2D MOFs have more abundant accessible active sites.^[^ ^58^ ^]^ Third, the substrates and products can diffuse quickly thanks to the 2D structure with nanometer thickness, contributing to high reaction rates.^[^ ^59^, ^60^ ^]^ More importantly, because of the macrocyclic rigid structure of Por/Pc and the fixed direction angle, it is easier to design and predict the structure of 2D Por/Pc‐MOF than traditional MOF. Up to now, 2D Por/Pc‐MOF nanosheets are usually constructed by top‐down and bottom‐up methods (**Figure** **2**).

The top‐down method refers to exfoliating the layer structures of MOFs, mainly including sonication exfoliation, mechanical exfoliation, chemical exfoliation, and so on.^[^ ^61^ ^]^ Among them, it is convenient to break the interlayer interactions within MOFs through sonication exfoliation, which is extensively applied in top‐down methods. As shown in Figure 2a, the ultrathin 2D Cu‐MOFs nanosheets were developed via sonication exfoliation of the Cu‐MOFs membrane, which developed from the interfacial reaction between the TCPP ligand dissolved in the organic phase and Cu^2+^ from aqueous solution.^[^ ^62^ ^]^ Atomic force microscopy (AFM) image of the Cu‐MOFs nanosheets displayed the accordant thickness of ≈3.4 nm. Then the 2D Cu‐MOFs decorated Au NPs display evident catalytic reduction activity for the 4‐nitrophenol and steady catalytic efficiency after several recycles because the 2D nanosheets not only avoid the aggregation of Au NPs but also generate a synergetic effect to improve the catalytic activities.

However, in the vertical direction of MOFs, if there are strong interactions (such as hydrogen‐bond interaction, *π*‐*π* stacking interaction, and van der Waals force), only sonication exfoliation cannot exhibit a significant effect. Other top‐down synthetic strategies have been tried to employ for the exfoliation of layer‐MOFs.^[^ ^63^ ^]^ Recently, ball milling as one of the mechanical exfoliation methods been used to exfoliate the bulk MOF (Ni_2_[CuPc(NH)_8_]), which breaks the interactions existing in layer‐structured materials via external mechanical forces (Figure 2b).^[^ ^64^ ^]^ In the process of ball milling exfoliation, the addition of NaCl inserts into the layers serves as a controlling agent, which can effectively reduce the shear forces and mildly exfoliate the bulk MOF. In addition, the presence of NaCl can contribute to larger‐sized nanosheets contrasted with the controls obtained in the absence of it. The X‐ray diffraction (XRD) pattern of Ni_2_[CuPc(NH)_8_] showed the shift of (002) peak from 27.51° (bulk crystals) to 26.74° (nanosheets), indicating the generation of swollen layers after exfoliation via the method of ball milling. Meanwhile, the ball milling method offer a high yield reaching 40–50% through exfoliating the well‐defined bulk MOFs into nanosheets.

In addition, the intercalation/chemical exfoliation method has also been known as an effective strategy to cripple layer interactions and improve the sonication processes. For example, the intercalation/chemical exfoliation method was developed to exfoliate layered MOF into ultra‐thin 2D MOF nanosheets (Figure 2c).^[^ ^65^ ^]^ In this work, a chemically unstable dipyridyl molecule, 4,4′‐dipyridyl disulfide (DPDS), was inserted into the layered MOFs to construct novel intercalated MOFs. Subsequently, the ultrathin 2D MOF nanosheets were developed by chemical exfoliation method using trimethylphosphine to selectively break the disulfide bond in DPDS. Because of the weakened interlayer‐interaction, the layered MOFs were exfoliated under gentle stirring to become ultrathin (≈1 nm) 2D MOF nanosheets with higher yield (≈57%).

The bottom‐up methods consider that 2D Por/Pc‐MOF nanosheets are directly synthesized from organic ligands and metal salts, including the direct solvothermal method, surfactant‐assisted method, and interfacial method. For instance, the crystalline structures and morphologies of 2D Por/Pc‐MOFs can be regulated through the directed solvothermal synthesis method, including changing the reaction conditions such as reaction time, solvents, and temperature (Figure 2d).^[^ ^66^ ^]^ If the growth rate of MOF crystal along the vertical direction is much lower than that on edge, the crystal surface with lower growth rates will be easily exposed, which is in favor of getting 2D MOF nanosheets. However, the development of a general route for 2D Por/Pc‐MOF synthesis by the direct solvothermal method is still a major challenge, as the method depends heavily on the choice of reaction conditions and solvent.

So far, during bottom‐up methods, surfactant‐assisted synthesis has become more widely used for the preparation of uniform and ultra‐thin 2D MOF nanosheets because the surfactants with amphiphilic nature can bind to the surface of MOFs at one end and repel other MOFs at the other end.^[^ ^69^ ^]^ For example, a serious of TCPP‐based ultrathin 2D MOFs were first prepared via the surfactant‐assisted synthesis method, which has a uniform thickness of less than 10 nm (Figure 2e). In the reaction, one TCPP ligand was connected with four Zn_2_(COO)_4_ secondary building units (SBUs) to form the 2D layered sheets. Zinc ions metalized TCPP ligands to form a Zn‐TCPP structure, in which the zinc atom was coordinated at the center of the Por macrocycle. Furthermore, the Zn‐TCPP and Zn_2_(COO)_4_ SBUs were stacked in the form of AB packing to obtain the final 2D MOF.^[^ ^67^ ^]^ During the synthetic process, PVP surfactant was used as a topology modulator, which can limit the generation of a 3D bulk structure by bonding to the surface of the MOFs. The low contrast of the 2D nanosheet in the transmission electron microscopy (TEM) image proved its ultra‐thin property.

As shown in Figure 2f, the interfacial synthesis method is broadly employed in the development of 2D MOFs too. The advantage of this bottom‐up approach is that organic ligands and metal nodes can only grow in a restricted area of the two‐phase interface to form nanosheets.^[^ ^68^ ^]^ Meanwhile, the desired MOF nanosheets can also be efficiently produced by choosing a reaction vessel with a large surface area. For example, the interfacial synthesis method is used to construct the CoTCPP–py–Cu nanosheets, which were prepared at the water/air interface. To be specific, CoTCPP and pyridine first spread among chloroform/methanol solution, and then the mixture solution was spread into CuCl_2_ aqueous solution. As a result, 2D CoTCPP‐py‐Cu was formed by the coordination reaction between CoTCPP and copper at the water/air interface after the volatilization of the chloroform/methanol solution. Especially, the monolayer nanosheets synthesized by the above method can be deposited on the substrate to form controllable thickness and layered structure nanosheets.^[^ ^70^ ^]^

However, there are some drawbacks for the top‐down methods. First, the synthesis of 2D MOF nanosheets via top‐down methods is only suitable for MOFs with layered structures. Besides, to a great extent, a few defects of top‐down methods restrict their universal applications, including difficult scale‐up, interlayer restacking, high energy consumption, and low yield.

Compared with top‐down methods, bottom‐up methods further investigated the crystallization kinetics and self‐assembly mechanism of MOF nanocrystals, which possess several advantages, including 2D MOF with a high yield, controllable thickness, and larger lateral size. However, the bottom‐up method always involves complicated procedures, which need to be further improved in the future.

#### 2.1.2 Synthesis of 3D Porphyrins/Phthalocyanines‐Metal–Organic Framework

**Figure 3:**
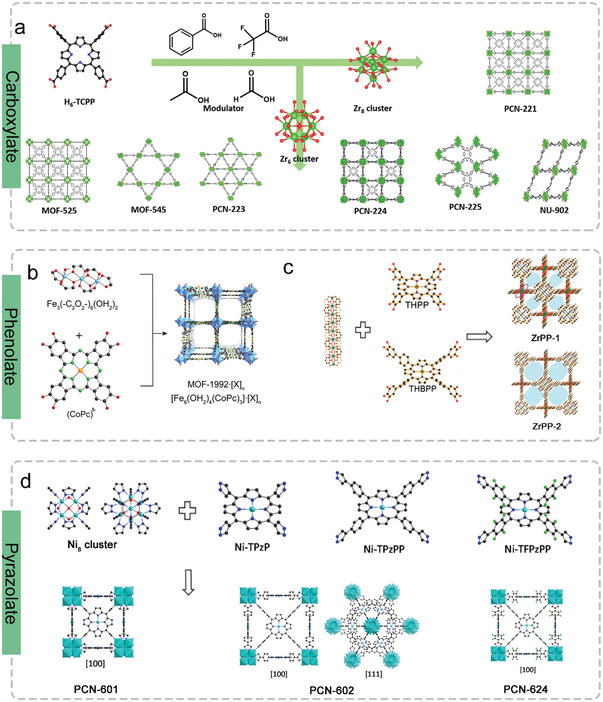
3D Por/Pc‐MOFs where Por/Pc is based on different functionalized groups coordinated with high‐valence metal ions/clusters. a) The syntheses of 3D MOFs based on Por functionalized pyrazolyl and Ni_8_ cluster. Reproduced with permission.^[^ ^73^ ^]^ Copyright 2021, The Korean Institute of Chemical Engineers. The syntheses of 3D b) Pc‐ and c) Por‐ phenolate MOFs. b) Reproduced with permission.^[^ ^76^ ^]^ Copyright 2019, American Chemical Society. c) Reproduced with permission.^[^ ^77^ ^]^ Copyright 2017, WILEY‐VCH. d) The syntheses of 3D MOFs based on pyrazolyl‐Pors. Reproduced with permission.^[^ ^55^ ^]^ Copyright 2021, The Royal Society of Chemistry.

Compared to 2D Por/Pc‐MOF, 3D Por/Pc‐MOFs account for the majority of the Por/Pc‐MOFs domain in the field of electrocatalysis because of the following advantages.^[^ ^71^ ^]^ First of all, Por/Pc‐MOFs with typical 3D topologies possess higher accessible active sites to improve the current density and reduce the aggregation of Por/Pc molecules, which can maximize the volume activity of the electrocatalyst.^[^ ^72^ ^]^ Second, the large surface areas in Por/Pc‐MOFs can effectively reduce the charge transfer resistance by forming seamless contact with electrodes, allowing for enhanced electrolyte penetration to accelerate the bubble release rates. Unlike the above‐mentioned synthetic methods of 2D Por/Pc‐MOFs, solvothermal is the common way to prepare 3D Por/Pc‐MOFs. Generally, the key to realizing 3D topology is to combine the 4‐connected nodes of Por/Pc square planar with the metal ions/clusters nodes owing abundant coordinated sites to extend the dimension of the overall structure further. Recently, a series of 3D Por/Pc‐MOFs in different topologies have been synthesized via the reaction between high‐valence metal ions/clusters (Zr^4+^, Hf^4+^, Al^3+^, Fe^3+^, Cr^3+^) and Por/Pc with the functionalized groups (carboxy, phenolic hydroxyl, pyrazolyl, sulfonic acid, and so on) under the similar solvothermal conditions. Due to the addition of a modulator (chemical additive), different MOFs are produced by controlling the crystallization process, benefiting from their flexible coordination bond.^[^ ^73^, ^74^, ^75^ ^]^ In this part, we will introduce 3D Por/Pc‐MOFs where Por/Pc is based on different functionalized groups coordinated with high‐valence metal ions/clusters (**Figure** **3**).

In a typical synthetic process, 3D Por/Pc‐MOFs are usually developed by a solvothermal reaction of metal salts and organic ligand precursors, which may take several hours to days to synthesize them in *N*,*N*′‐dimethylformamide (DMF) deprotonating solvent or hydrothermally in water. As shown in Figure 3a, TCPP with different functionalized carboxyl groups can be linked with zirconium‐oxo clusters to form several 3D Por/Pc‐MOFs with different topologies, including those constructed by Zr_6_ cluster (MOF‐525,^[^ ^78^ ^]^ MOF‐545,^[^ ^78^ ^]^ NU‐902,^[^ ^79^ ^]^ PCN‐223,^[^ ^80^ ^]^ PCN‐224,^[^ ^45^, ^81^ ^]^ and PCN‐225^[^ ^82^ ^]^) and Zr_8_ cluster (PCN‐221^[^ ^83^ ^]^). However, the high charge density of Zr^4+^ can polarize the Zr‐O bond to prevent the reaction between the Zr cluster and TCPP, resulting in the low crystallinity of MOF. It is necessary to introduce an acid modulator during the synthesis to compete against the TCPP building block to coordinate with the Zr cluster, which can slow the nucleation rate to overcome such an issue.^[^ ^84^ ^]^ Instead of carboxyl‐modified Por/Pc building blocks, Por/Pc with polyphenol groups can act as polydentate chelating ligand binding to metal ions, benefit forming the steady coordination site in 3D MOF, leading to enhanced stability. For instance, in the DMF/water/MeOH mixed solution, the 3D MOF was obtained by reacting catechol Co‐phthalocyanine (CoTCatPc) with FeCl_2_ under solvothermal conditions (Figure 3b).^[^ ^76^ ^]^ An anionic 3D framework with a roc topology was generated via each Pc connecting four different iron trimers.

Later on, two polyphenol‐based Pors (5,10,15,20‐tetrakis(3,4,5‐trihydroxybiphenyl)porphyrin (THPP) and extend 5,10,15,20‐tetrakis(3,4,5‐trihydroxybiphenyl)porphyrin) (THBPP) are used to construct two similar structure Zr‐based 3D MOFs (named ZrPP‐1 and ZrPP‐2, respectively) (Figure 3c).^[^ ^77^ ^]^ The robustness of such material has been verified again by immersing it in 20 m NaOH for a long time. In addition, pyrazolate‐based Por (such as TPzP, TPzPP, TFPzPP) as nitrogen donor ligands are also utilized to prepare 3D Por‐MOFs (such as PCN‐601,^[^ ^85^ ^]^ PCN‐602,^[^ ^86^ ^]^ and PCN‐603^[^ ^87^ ^]^) in the pure DMF system under moderate solvothermal environment (75 to 130 °C) (Figure 3d).^[^ ^55^ ^]^

In addition, polyoxometalates‐based Por/Pc‐MOFs (POM‐POFs) are a special class of 3D Por/Pc‐MOFs, in which POMs containing abundant transition metal elements with high oxidation state can easily transfer electrons during reversible redox reactions without changing their metal‐oxo cluster structures, thus, more excellent electrochemical performance will be given by the synergistic combination of POMs and M‐Por/Pc building block.^[^ ^88^, ^89^, ^90^, ^91^ ^]^ For example, a Co‐PMOF, which is constructed by Zn‐*ε*‐Keggin POM and Co‐TCPP building block via the solvothermal method, exhibits excellent electrochemical CO_2_ reduction performances. Because under the exertion of the electric field, the oriented electronic transportation channel is constructed by the direct communication of Zn‐*ε*‐Keggin and Co‐TCPP, thus benefiting the multiple electrons transfer process in CO_2_RR.^[^ ^92^ ^]^

### 2.2 Synthesis of Porphyrins/Phthalocyanines‐Covalent Organic Framework

**Scheme 3:**
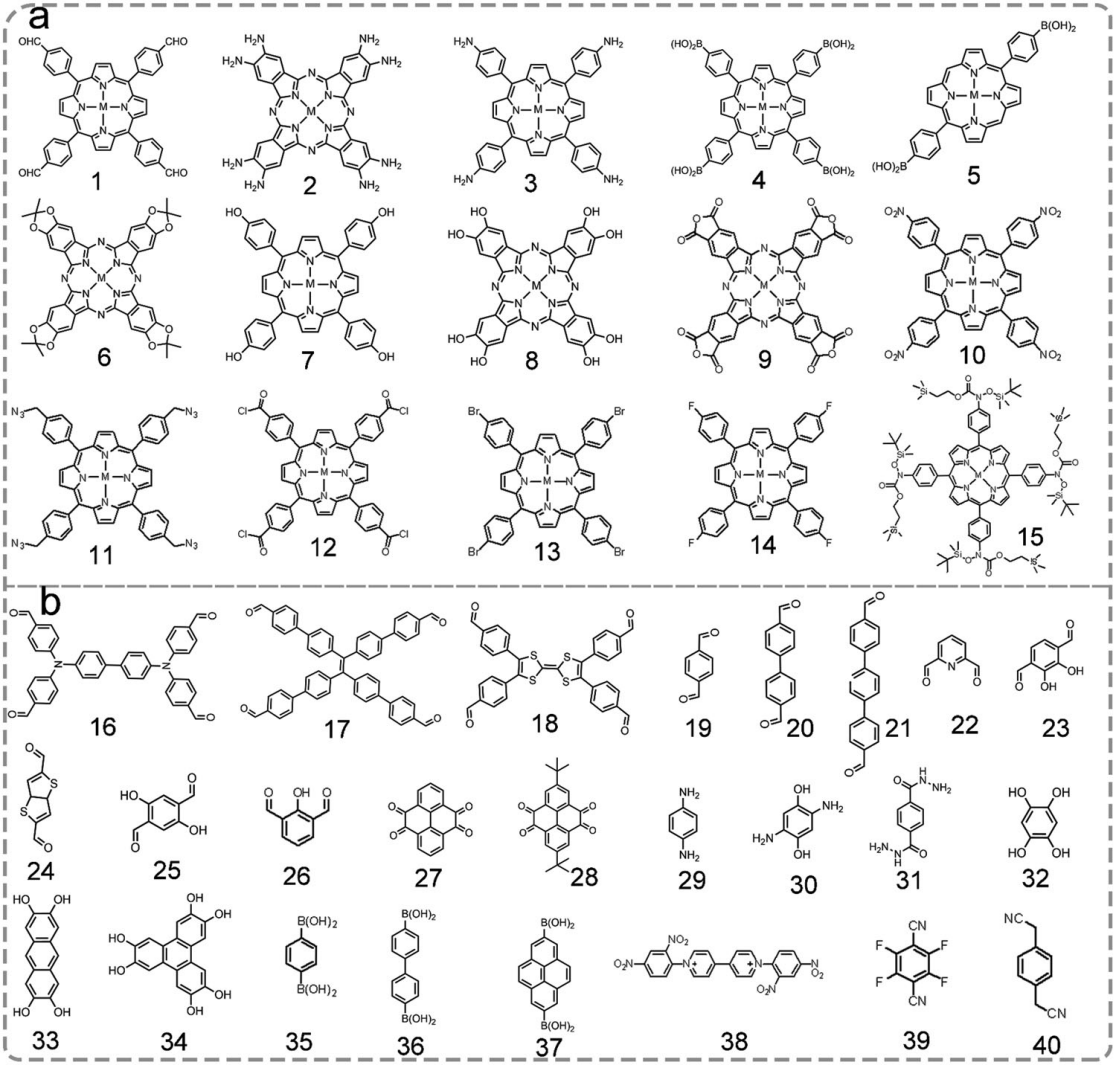
The COFs’ molecular structure of a) M‐Por/M‐Pc building blocks and b) organic linkers. a) 5,10,15,20tetrakis(4‐benzaldhyde)porphyrin (TFPP, 1), 2,3,9,10,16,17,23,24‐phthalocyanine octadecylamine (OAPc, 2), 5,10,15,20‐tetra(*p*‐aminophenyl)porphyrin (TAPP, 3), 5,10,15,20‐tetrakis(4‐(dihydroxyboryl)phenyl) porphyrin (TDHB, 4), 5,15‐bis(4‐boronophenyl)‐porphyrin (BBPP, 5), phthalocyanine tetra(acetonide) (6), 4,4′,4″,4‴‐(21H,23H‐porphine‐5,10,15,20‐tetrayl)tetrakis‐Phenol (T(OH)_4_PP, 7), 2,3,9,10,16,17,23,24‐octahydroxyphthalocyanine (8OH‐Pc, 8), tetraanhydrides of 2,3,9,10,16,17,23,24‐octacarboxyphthalocyanine (TAPc, 9), 5,10,15,20‐tetrakis(4‐nitrophenyl)porphyrin (TNPP, 10), 5,10,15,20‐tetrakis(*α*‐azido‐*p*‐tolyl) porphyrin (TPP‐4N_3_, 11), TCPP chloride (12), meso‐tetra(*p*‐bromophenyl)porphyrin (13), meso‐tetra(*p*‐fluorophenyl)porphyrin (F‐Por, 14), tetrakis(arylhydroxylamine)porphyrin (15). b) 4,4′,4″,4‴‐([1,1′biphenyl]‐4,4′‐diylbis(azanetriyl))‐tetrabenzaldehyde (BDTA, 16), 1,10,2,20‐tetrakis(4‐formyl‐(1,1′‐biphenyl))ethene (TFBE, 17), 2,3,6,7‐tetra(4‐formylphenyl)tetrathiafulvalene (4‐formyl‐TTF, 18), (19) terephthaldehyde (TA, 19), biphenyl‐4,4′‐dicarboxaldehyde (BPDA, 20), 4,4′,4″,4‴‐([1,1′biphenyl]‐4,4′‐diylbis(azanetriyl))tetrabenzal‐dehyde (BDTA, 21), 2,6‐pyridinedicarboxaldehyde (PCBA, 22), 2,3‐dihydroxyterephthalaldehyde (2,3‐Dha, 23), thieno[3,2‐*b*]thiophene‐2,5‐dicarboxaldehyde (TT, 24), 2,5‐dihydroxyterephthalaldehyde (DA, 25), 2,6‐diformylphenol (DFP, 26), pyrenetetraone (TOPyr, 27), *tert*‐butylpyrene−tetraone (^t^Bu‐PT, 28), *p*‐phenylenediamine (PD, 29), 2,5‐dihydroxy‐*p*‐benzaldehyde (DHA, 30), 2,5‐diethoxyterephthalohydrazide (DETH, 31), 1,2,4,5‐tetrahydroxybenzene (THB, 32), 2,3,4,5‐tetrahydroxy anthracene (THAn, 33), 2,3,6,7,10,11‐hexahydroxytriphenylene (34), 1,4‐phenylenebis(boronic acid) (PBBA, 35), 4,4′‐biphenyl bisboronic acid (BBA, 36), pyrene‐2,7‐diboronic acid (PDBA, 37), tetranitroviologen (TNV, 38), tetrafluorophthalonitrile (TFPN, 39), 1,4‐phenylenediace‐tonitrile (PDAN, 40).

Unlike the MOF structure, which is constructed through the coordination between metal ions/clusters and Por/Pc building blocks, COF needs to be formed by the covalent linkage of organic ligands. Since the first COF was synthesized by Yaghi and his colleagues, electron‐enrichment systems such as Pc, Por, and pyrene building blocks have often been applied to develop novel COF materials.^[^ ^32^, ^93^, ^94^ ^]^ Generally, the rigid structure of mentioned electron‐enrichment systems is conducive to the topological structure design of COFs.^[^ ^95^, ^96^ ^]^ Particularly, Por/Pc can adjust their catalytic ability through the doping and substituent effects of various metals, which make them become common building blocks of COFs. Considering the covalent connectivity of COF in different dimensionalities, Por/Pc‐COFs can be divided into 2D layered structures or 3D networks. Building block structures, linkages, and synthesis methods are the key points in designing and synthesizing COFs to achieve targeting properties, cost‐effective synthesis, tunable porosity, and different physicochemical properties.^[^ ^97^ ^]^ **Scheme** **3** shows the M‐Por/M‐Pc building blocks and organic linkers commonly used in the synthesis of Por/Pc‐COFs. Because of the modular nature of COFs, Por/Pc can be evenly dispersed throughout the COFs. Therefore, the metal sites coordinated in the macrocycle can be effectively isolated at the molecular level. The Por/Pc‐COFs can expose more accessible metal active sites, which may improve catalytic activity.^[^ ^98^, ^99^, ^100^ ^]^ In this part, we clearly discuss the synthesis of Por/Pc‐COFs, which will be discussed as 2D layered structures or 3D networks from the perspective of linkage.

#### 2.2.1 Synthesis of 2D Porphyrins/Phthalocyanines‐Covalent Organic Framework

In recent years, 2D Por/Pc‐COF has been extensively studied because of the excellent superiority of various and designable structures. Meanwhile, in 2D Por/Pc‐COF, the building blocks and organic linkers are connected in the formation of covalent bonds and construct aligned p‐columns layered structures that can facilitate carrier transport via pre‐organized pathways, making them a promising candidate for electrocatalysis.^[^ ^101^ ^]^ Contrary to the 3D structure that dominates Por/Pc MOF, Por/Pc COF is controlled by 2D species because there are more available building blocks with controllable active sites, geometric structure, and size for easily constructing 2D Por/Pc‐COFs.^[^ ^37^ ^]^ To be specific, the planar quadrangle geometry of Por/Pc and the existence of template polymerization have an influence on the formation; thus, when compared with the synthesis of 3D COFs, 2D structures is easier. Until now, dynamic covalent chemistry can construct reversible covalent linkages to form an ordered framework, which has been widely used in synthesizing 2D Por/Pc‐COF.^[^ ^46^, ^102^ ^]^ In this part, we will mainly debate different building blocks and linkages of 2D Por/Pc‐COF. Some linkages (such as boronate, imine, ether, imide, *sp* ^2^‐carbon, azo, and so on) and their characteristics are widely used for constructing 2D Por/Pc‐COFs, and **Table** **1** shows specific comparisons.^[^ ^39^, ^40^, ^103^, ^104^, ^105^, ^106^, ^107^, ^108^, ^109^, ^110^, ^111^ ^]^

**Table 1:** Summary of linkages, building blocks, and characteristics of several typical 2D Por/Pc‐COFs

COF	Linkages	Characteristics	Por/Pc building block	Organic linker	Ref.
Pc‐PBBA	Boronate 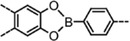	1) Excellent crystallinity; 2) Excellent thermal stability; 3) Poor chemical stability.	Phthalocyanine tetra(acetonide)	PBBA	[103]
ZnP‐COF			TDHB	THB	[104]
COF‐66			TBPP	THAn	[105]
COF‐366	Imine 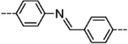	1) Good crystallinity; 2) Excellent thermal stability; 3) Good chemical stability.	TAPP	TA	[105]
JUC‐605			TAPP	TPA	[40]
RICE‐1			TAPP	ETTA	[106]
COF‐DC‐8			OAPc	TOPyr	[107]
2D CPF	Azo 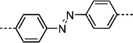	1) Good crystallinity; 2) Moderate thermal stability; 3) Good chemical stability.	TAPP	TNPP	[108]
COF‐Pors	Ether 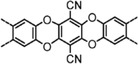	1) Moderate crystallinity; 2) Excellent thermal stability; 3) Excellent chemical stability.	T(OH)_4_PP	F‐Por	[109]
Pc‐TFPN COF			Pc‐8OH	TFPN	[39]
Pc‐PI‐COF‐1	Imide 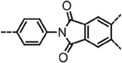	1) Good crystallinity; 2) Excellent thermal stability; 3) Good chemical stability.	TAPc	PD	[110]
Por‐sp^2^c‐COF	sp^2^‐carbon 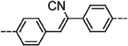	1) Moderate crystallinity; 2) Moderate thermal stability; 3) Excellent chemical stability.	TFPP	PDAN	[111]

**Figure 4:**
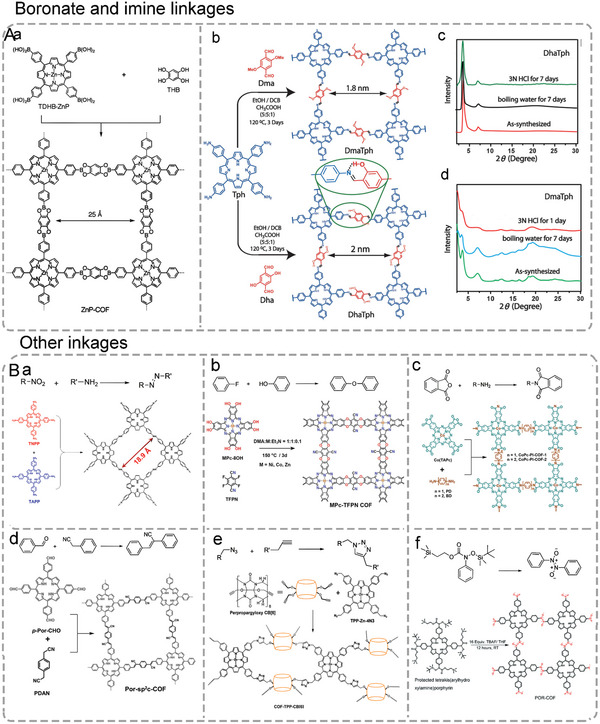
Scheme of the synthetic methods of different 2D Por/Pc‐COF. A) Boronate and imine linkages of Por/Pc‐COFs. a) Synthesis of boronate‐linked ZnP‐COF. Reproduced with permission.^[^ ^104^ ^]^ Copyright 2011, The Royal Society of Chemistry. b) Synthesis of imine‐linked DhaTph and DmaTph. c,d) Powder X‐ray diffraction (PXRD) pattern of DhaTph and DmaTph, respectively. Reproduced with permission.^[^ ^112^ ^]^ Copyright 2010, Macmillan. B) Other linkages of 2D Por/Pc‐COFs. a) Reproduced with permission.^[^ ^108^ ^]^ Copyright 2021, Elsevier. b) Synthesis of dioxin‐linked MPc‐TFPN COF. Reproduced with permission.^[^ ^39^ ^]^ Copyright 2020, Wiley‐VCH. c) Synthesis of imide‐linked CoPc‐PI‐COF. Reproduced with permission.^[^ ^110^ ^]^ Copyright 2021, American Chemical Society. d) Synthesis of *sp* ^2^‐carbon‐linked Por‐*sp* ^2^c‐COF. Reproduced with permission.^[^ ^111^ ^]^ Copyright 2019, Wiley‐VCH. e) Synthesis of azide‐alkyne linked COF‐TPP‐CB[6]. Reproduced with permission.^[^ ^113^ ^]^ Copyright 2021, American Chemical Society. f) Synthesis of azodioxy‐linked MPc‐TFPN COF. Reproduced with permission.^[^ ^114^ ^]^ Copyright 2016, The Royal Society of Chemistry.

As a typical example, the condensation of boric acid and diol to form borate is a common reaction in organic synthesis reactions, which is also an efficient strategy for synthesizing advanced structures of 2D Por/Pc‐COF. As shown in **Figure** **4**A‐a, the first boronate‐linked Por‐COF was developed by Jiang and colleagues, which was started from 1,2,4,5‐tetrahydroxybenzene (THB) and zinc(II) 5,10,15,20‐tetrakis(4‐(dihydroxyboryl)phenyl) porphyrin (TDHB‐ZnP).^[^ ^104^ ^]^ Similarly, the Pc‐PBBA COF, as the first Pc‐COF, was developed by 1,4‐phenylenebis(boronic acid) and phthalocyanine tetra(acetonide).^[^ ^103^ ^]^ The reversibility of the condensation reaction consisting of borated bonds shows the highly ordered structure and high thermal stability of these boric acid‐based 2D COFs. However, the Por/Pc‐COFs based on boronated linkages are easy to hydrolyze in humid environments or water, which limits their applications in aqueous catalysis systems.

Due to the weak solution stability of boronate‐linked 2D Por/Pc‐COFs, imine‐linked 2D Por/Pc‐COFs constructed by amine and aldehyde were designed.^[^ ^93^, ^115^ ^]^ Compared with boronate‐linked 2D Por/Pc‐COFs, the existence of extended *π*‐conjugation structure in the imine‐linked 2D COFs result in higher chemical stability and a wider scope of applications.^[^ ^116^ ^]^ Due to the limited reversibility of amine and aldehyde condensation reaction, the crystallinity of imine‐linked 2D Por/Pc‐COFs is lower than that of boronate‐linked 2D Por/Pc‐COFs. For example, the first reported 2D Por‐COF, named COF‐366, was constructed by the reversible condensation reaction of aldehyde from terephthaldehyde (TA) and amine from tetra (*p*‐amino‐phenyl) porphyrin (TAPP).^[^ ^117^, ^118^ ^]^ To further improve the crystallinity and chemical stability of 2D Por‐COFs, DhaTph‐COF was constructed via TAPP and Dha, which introduced ‐OH functional groups of linker near amide bonds to form intramolecular hydrogen bonds, thus protecting the internal structure of imine‐linked Por‐COF (Figure 4A‐b).^[^ ^112^ ^]^ As shown in Figure 4A‐c,d, the presence of hydrogen bonds in DhaTPh‐COF enhances the crystallinity of conjugated framework, chemical stability, and porosity compared to DmaTph without the intramolecular hydrogen bond.

In addition, as shown in Figure 4B, many other covalently connected 2D Por/Pc‐COFs have been developed over time, such as azo‐linked (Figure 4B‐a), ether‐linked (Figure 4B‐b), imide‐linked (Figure 4B‐c), *sp* ^2^‐carbon‐linked (Figure 4B‐d), azide–alkyne linked (Figure 4B‐e), and azodioxy‐linked (Figure 4B‐f) 2D Por/Pc‐COFs. Recently, the azo‐linked 2D Por‐COF named CPF has been constructed by solvothermal reactions starting with tetrakis‐(4‐nitro‐phenyl)‐porphyrin (TNPP) and tetrakis‐(4‐amino‐phenyl)‐porphyrin (TAPP), which exhibit good crystallinity, moderate thermal stability, and good chemical stability (Figure 4B‐a).^[^ ^108^ ^]^ Meanwhile, an emerging kind of 2D Por/Pc‐COFs with ether (—C—O—C—) linkage is reported, which provides outstanding chemical stability to this 2D COF due to the existence of ether linkage (Figure 4B‐b).^[^ ^39^ ^]^ Although in extreme chemical environments such as strong acids, strong bases, boiling water, and oxidizing as well as reducing conditions, these ether‐linked COFs remain stable, greatly expanding their application environment.

Compared with the as‐mentioned imine‐linked 2D COFs in Figure 4A‐b, 2D COFs with imide linkage have higher stability, especially in acidic conditions (Figure 4B‐c).^[^ ^110^ ^]^ However, because of the key criterion for constructing COFs, the reversibility of the condensation reaction has been a barrier resulting in the reported imide‐linked 2D COF rarely. As for the *sp* ^2^‐carbon‐linked 2D COFs, they had been shown high stable in acids, bases, and organolithium reagents due to the high reversibility of C = C linkage imparted by nitriles attached to the C = C bond. Wang and coworkers reported a novel *sp* ^2^‐carbon linked Por‐COF (Por‐*sp* ^2^c‐COF) starting with 1,4‐phenylenediacetonitrile (PDAN) and 5,10,15,20‐tetrakis(4‐benzal dehyde)porphyrin (*p*‐Por‐CHO) (Figure 4B‐d). As one of the most common types in 2D Por‐COFs, the layered Por‐*sp* ^2^C‐COF has square channels with Por units located at the nodes of square skeletons and linked by C = C bonds.^[^ ^111^ ^]^ Besides the linkages mentioned above, there are a few other linkages, such as azide–alkyne linked^[^ ^113^ ^]^ (Figure 4B‐e) and azodioxy‐linked^[^ ^114^ ^]^ (Figure 4B‐f) 2D Por/Pc‐COFs. However, the closely packed 2D layered structures of COFs, especially the overlapping stacking through strong *π*–*π* interactions, will inevitably reduce the utilization of active sites, resulting in a low electrocatalytic activity.

#### 2.2.2 Synthesis of 3D Porphyrins/Phthalocyanines‐Covalent Organic Framework

Compared with 2D COFs, 3D COFs possess the whole 3D structure connected covalently, whereas the covalent bonds of 2D COFs exist only in conjugated 2D flakes. The combination of Por/Pc into 3D COFs makes all Por/Pc units within the framework accessible so that 3D Por/Pc‐based COFs have remarkably large surface areas and numerous open sites, which could expose the active sites to the pore wall surface and allow them to be accessed for substrates, resulting higher catalytic activities.^[^ ^36^ ^]^ To date, the reports on the design and synthesis of 3D Por/Pc‐COFs are only a few number types.^[^ ^119^ ^]^ Constructing 3D COFs remain challenging because the driving force for synthesizing 3D COFs depends only on covalent bonds. Thus, the development of stable 3D Por/Pc‐COFs is urgently needed. It is still an exciting topic to develop novel 3D Por/Pc‐COFs from 2D Por/Pc‐COFs chemical reactions. In this part, we mainly introduce the current methods of synthesizing 3D COFs, such as imine‐linked and boronate‐linked methods via [4+4] condensation reaction of tetrahedral and quadrilateral building blocks.

**Figure 5:**
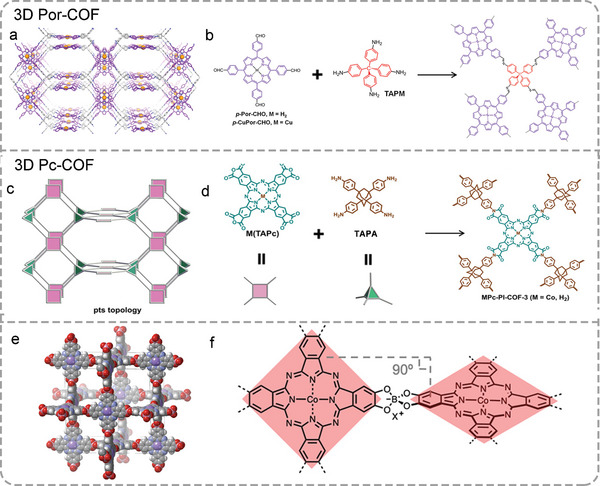
Illustration of the synthesis of 3D Por/Pc‐COF. a) Simulated structures of 3D‐Por‐COF. b) Synthesis of the 3D‐MPor‐COF (M = H_2_ or Cu). Reproduced with permission.^[^ ^120^ ^]^ Copyright 2017, American Chemical Society. c) Illustration of pts topology. d) Synthesis of MPc‐PI‐COF‐3. Reproduced with permission.^[^ ^37^ ^]^ Copyright 2021, Wiley‐VCH. e) Single crystal structure of SPB‐COF‐DBA. f) Synthesis of the SPB‐COF‐DBA. Reproduced with permission.^[^ ^47^ ^]^ Copyright 2021, American Chemical Society.

As shown in **Figure** **5**a,b, the two 3D Por‐COFs linked by imine are synthesized through [4+4] imine co‐condensation reaction of tetrahedral tetra(*p*‐aminophenyl)methane (TAPM) with an amino group and quadrilateral monomer 5,10,15,20‐Tetrakis(4‐benzaldehyde)porphyrin copper (*p*‐CuPor‐CHO) with aldehyde group, which are different from the traditional co‐condensation of tetrahedral node and linear component for synthesizing 3D COF.^[^ ^120^ ^]^ Another imide‐linked 3D Pc‐COFs is synthesized by 1,3,5,7‐tetra(4‐aminophenyl)adamantine (TAPA) and 2,3,9,10,16,17,23,24‐octacarboxyphthalocyanine tetraanhydride M(TAPc) (M = Co, H_2_), resulting in complicated interpenetrated pts networks (Figure 5c,d).^[^ ^37^ ^]^ Recently, a novel 3D Pc‐COF linked by boronate ester with a noninterpenetrated nbo topology has been reported, namely, SPB‐COF‐DBA, which is produced by the reaction of cobalt(II) 2,3,9,10,16,17,23,24‐octahydroxyphthalocyaninato ((OH)_8_PcCo) and tetrahedral spiroborate (SPB) in *N*,*N*‐dibutylformamide solvent at 120 °C for 3 days (Figure 5e,f).^[^ ^47^ ^]^ Neighboring CoPc units are linked by the SPB linkers providing the 90° dihedral angles. The high‐resolution transmission electron microscope images of SPB‐COF‐DBA show a well‐ordered crystal lattice and high crystallinity.

## 3 Electrochemical Applications of Porphyrins/Phthalocyanines‐Based Organic Frameworks

During the past decades, extensive efforts have been devoted to developing earth‐abundant, low‐cost, and efficient electrocatalysts for clean energy. POFs offer various remarkable characteristics taking advantage of the integration of Por/Pc into frameworks, such as distinguished electrochemical properties, stability, porous structure, and multi‐functions of the Por/Pc molecular building blocks.^[^ ^121^ ^]^ These features are in favor of applications of POFs in electrochemistry, which have drawn more and more attention. Furthermore, transition metal coordinated POFs have the characteristics of structural flexibility and diversity of building units and can facilely introduce various heteroatoms and metal species into the porous structure. Nowadays, numerous emerging electrochemical applications, such as CO_2_ reduction flow cells (CO_2_RR), fuel cells (involving ORR), rechargeable Zn–air batteries (ORR and OER), and electrolytic water‐splitting cells (including HER and OER) have been developed for POFs.^[^ ^122^ ^]^

In the above sections, we have comprehensively discussed how to design the structure of POFs. In this part, we will summarize that the precision control on the coordination microenvironments of POFs‐based electrocatalysts can be accomplished by the diverse modulating strategies, including tailoring the microenvironment of the catalytic center, tuning topology, and doping hybrid system. In the following, we review the representative advancement in electrochemical applications of POFs, including CO_2_RR, ORR, HER, and OER.^[^ ^123^, ^124^, ^125^ ^]^

### 3.1 Modulating the Electrocatalytic Activity of Porphyrins/Phthalocyanines‐Based Organic Frameworks

Apart from the variety of linkers composed of POFs and metal active sites, there are many other factors, such as topology, and hybrid systems, have been proposed to make the most of the unique characteristics of POFs. Thus, for further promotion of the electrocatalytic activity of POFs, some modulation strategies, such as tailoring coordination microenvironment, tuning topology, and adopting the hybrid system, have been proposed to further optimize the electrocatalytic performance through exposing more active catalytic sites, facilitating the mass transfer, and improving the electroconductivity of POFs. The modulation strategies and catalytic effects have been summarized in **Table** **2**. Meanwhile, we will describe the corresponding modulating strategies and catalytic effects of the POFs‐based electrocatalysts in detail with specific examples.

**Table 2:** The modulating strategies and catalytic effects of POFs structures

Modulation strategies	Schematic diagrams	Common methods	Augmented catalytic effects
Coordination microenvironment	Metal atom center 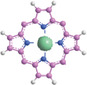 Substituent groups 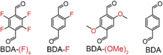 Heteroatom 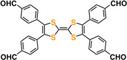	1) Active metal center: Fe, Co, Ni, Cu; 2) Electron withdrawing groups; 3) Heteroatom doping: S, N, P, etc.	1) Enhance intermolecular electron transmission efficiency; 2) Optimize the framework for high activity and selectivity; 3) Construct an oriented electron transmission pathway with M‐Por/Pc.
Topology tuningref. [36]	Small pore size 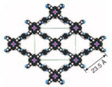 Large pore size 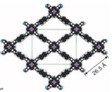 2D structure 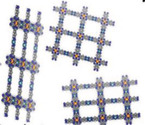 3D structure 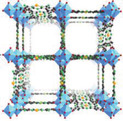	1) Pore size: Ligands of different sizes 2) Dimensionality 2D nanosheet: Top‐down method/ bottom‐up method 3D nanostructure MOF: Hydrothermal method, COF: [4+4] concentration	1) Higher electrochemical accessibility of the catalytic M‐Por/Pc active sites; 2) Higher capacity of gas adsorption inside the framework. 1) Expose larger surface area and more accessible active sites; 2) Promote the interaction with intermediate and substrate molecules. 1) Increase the available active sites; 2) Enhance the current density; 3) Reduce the aggregation of molecular building blocks
Hybrid system	Conductive support 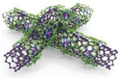	Graphene/CNT/FTO	Facilate the electron transfer from the electrode to M‐Por/Pc active sites.

#### 3.1.1 Modulating Metal Centers

Since the central metal atoms will interact directly with the raw materials and intermediates of electrochemical catalysis, the regulation of the central metal atom will significantly affect its intrinsic catalytic activity. Therefore, selecting proper central metal atoms is one of the most direct and effective strategies to regulate the electrocatalytic performance of POFs. However, it is an extremely challenging task to select the most suitable central metal atom (which also exhibits the highest intrinsic catalytic activity) solely on the basis of experimental results. To address this plight, density functional theory (DFT) calculations as an emerging theoretical simulation are used to compare the intrinsic electrocatalytic activity of POFs with different central metal atoms.^[^ ^126^ ^]^

**Figure 6:**
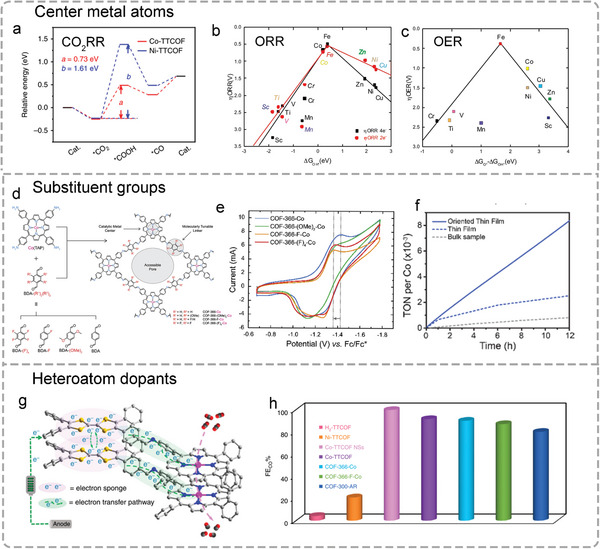
Regulation of the center metal atom. a) The relative CO_2_RR catalytic paths for TTCOF with Co center and Ni center. Reproduced with permission.^[^ ^38^ ^]^ Copyright 2020, Springer Nature. b) ORR and c) OER overpotentials as a function of adsorption energy for TM‐COFs. Reproduced with permission.^[^ ^127^ ^]^ Copyright 2017, WILEY‐VCH. d) The diagram of Co‐Por‐derived COFs. e) Cyclic voltammograms of Co‐Por derived COFs. f) Time‐dependent TON curves using the oriented thin films of COF‐366‐Co and other control materials. Reproduced with permission.^[^ ^118^ ^]^ Copyright 2017, American Chemical Society. g) Schematic diagram of the proposed CO_2_RR electrocatalytic mechanism of Co‐TTCOF. h) The electrocatalytic performance comparison of M‐TTCOFs with the recently reported works. Reproduced with permission.^[^ ^38^ ^]^ Copyright 2020, Springer Nature.

For instance, during CO_2_RR progress, the electroreduction of CO_2_ to CO involves three fundamental steps: 1) The formation of *COOH; 2) the formation of *CO; and 3) the CO desorption process. The formation of COOH* is the rate‐determining step (RDS) with a low energy barrier, which can be predicted on a precise M‐N‐C catalytic site through DFT. As shown in **Figure** **6**a, compared with Ni, Co‐TTCOF exhibits a significantly reduced Gibbs free energy for the formation of *COOH in the RDS, which is consistent with its higher electroreductive activity and selectivity.^[^ ^38^ ^]^ To expand more metal active centers, a variety of M‐Por‐MOF (M = Sc, Ti, V, Cr, Mn, Fe, Co, Ni, Cu, and Zn) were prepared, and their corresponding ORR and OER activity have been calculated by DFT calculations (Figure 6b,c).^[^ ^127^ ^]^ The results show that the type of transition metal determines whether the ORR process is 2e− transfer or 4e− transfer, and Co and Fe elements show the best theoretical electrocatalytic performance in the ORR process. Meanwhile, the results also exhibit that both Fe and Co show promising intrinsic OER activity.

In summary, based on the DFT calculations results and recent research of POFs applied in the field of electrochemistry, the results suggest that transition metals such as Fe, Co, Ni, and Cu are often coordinated with N in Por/Pc as active sites in POFs and largely determine the selectivity and activity of frameworks.

#### 3.1.2 Tailoring Microenvironment of Catalytic Center

The microenvironment of the catalytic center denoted the small specific and isolated chemicophysical environment around the metal atoms.^[^ ^128^ ^]^ Regulating the coordination microenvironments around the active sites is considered an effective strategy to modulate the bond interactions and interface microenvironments for enhancing the electrochemical catalytic performance, especially for the M‐N‐C structures of POFs catalysts. Because of the planar structure of the M‐Por/Pc, the skeleton of POFs is found to possess long‐range delocalized d/p electrons, which allows atoms far from each other to interact. In this section, we will summarize the synthetic strategies to tailor the coordination microenvironment of catalytic centers via the introduction of substituent groups and heteroatom dopants into POFs, which will affect the electronic structure of the center metal atom through long‐range delocalization.

The substituent groups with electronegative elements can have a direct electron‐withdrawing effect on the metal center, which results in significant differences in electronics and may improve the electrochemical catalytic activity of POFs. For example, a series of COFs from the COF‐366‐Co family with different BDA building block derivatives (such as BDA‐(F)_4_, BDA‐F, and BDA‐(OMe)_2_) as organic linkers were assembled to study the structure‐performance relationship of POF through modifying the linker with different electron‐withdrawing groups (Figure 6d).^[^ ^118^ ^]^ As shown in Figure 6e, the cyclic voltammograms (CV) show that the strongest electron‐withdrawing group modified COF‐366‐F‐Co catalyst exhibits the best CO_2_RR performance with the highest current density for CO formation (65 mA mg−1) when compared to other counterparts. Interestingly, the second‐highest electron‐withdrawing group (BDA‐(F)_4_) modified COF‐366(F)_4_‐Co exhibits the worst electrocatalytic performance. This can be owing to the high hydrophobicity of BDA‐(F)_4_ groups in COF‐366‐(F)_4_‐Co, which can reduce the catalytic sites’ contact with the electrolyte. At the same time, compared with the thin film and bulk sample, COF‐366‐Co oriented on the substrate acquired not only a low overpotential (550 mV) to achieve high current densities (65 mA mg−1) but also a high FE value (87%) through the reduction of CO_2_ to CO (Figure 6f).

N, S, P, and B, as the common dopant atoms, play a crucial part in enhancing the performances of catalysts in the field of electrochemical catalysis. Among the doping atoms mentioned above, S has been proven to improve the electrocatalytic performances of POFs in many studies. For example, M‐TAP (M = Ni or Co) was combined with 4‐formyl‐TTF (2,3,6,7‐tetra(4‐formyl phenyl)‐tetrathiafulvalene) with high electron mobility as a kind of electron donor to constructed a series of novel COFs (M‐TTCOFs).^[^ ^38^ ^]^ These novel COFs have the characteristics of stable and highly crystalline metal uniformly distributed, which exhibit the excellent electrocatalytic performance of CO_2_RR. Benefiting from the synergistic effect of TTF as an electron donor and Por as the electron acceptor, M‐TTCOFs have good electron transfer and electrocatalytic activity (Figure 6g). Moreover, Co‐TTCOF selectively converts CO_2_ to CO at −0.7 V, with a FE_CO_ value of 91.3%, which is also the highest among reported COFs (Figure 6h).

#### 3.1.3 Tuning Topology

**Figure 7:**
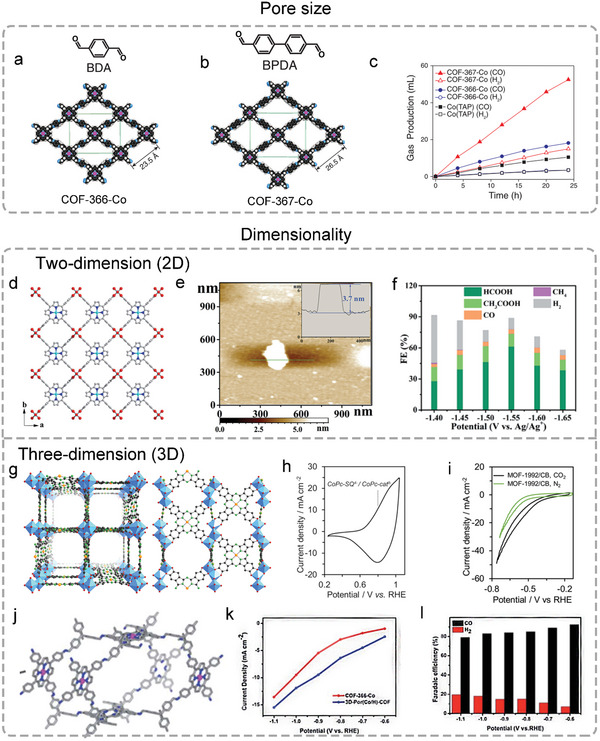
Topology tuning of POFs from pore size and dimensionality. Schematic of a) COF‐366‐Co and b) COF‐367‐Co. c) The volume of CO produced by electrolysis. Reproduced with permission.^[^ ^117^ ^]^ Copyright 2015, American Association for the Advancement of Science. d) Molecular packing of Cu_2_(CuTCPP) MOF view along the *c* axis. e) AFM image of Cu_2_(CuTCPP) MOF nanosheets. f) Faradaic efficiencies of Cu_2_(CuTCPP) nanosheets. Reproduced with permission.^[^ ^131^ ^]^ Copyright 2019, Royal Society of Chemistry. g) Structure of 3D MOF‐1922. h) CV for MOF‐1992/CB. i) CV for MOF‐1992/CB in KHCO_3_ solution. j) Schematic representation of 3D‐Por(Co/H)‐COF network. k) *j*_CO_. l) FE_CO_ of 3D‐Por(Co/H)‐COF in KHCO_3_ electrolyte. Reproduced with permission.^[^ ^36^ ^]^ Copyright 2022, Royal Society of Chemistry.

For electrocatalysts, elaborate topological design, such as pore size and dimensionality of POFs, need to be considered because it can expose more active sites and promote the mass transport of the electrocatalysts.^[^ ^129^ ^]^ The pore size and surface area of frameworks can affect exposed single active sites. Therefore, the electrocatalytic performance can be adjusted by changing the structure of the linkers to obtain POFs with different hole sizes.^[^ ^118^, ^130^ ^]^ For example, two Por‐COFs (COF‐366‐Co and COF‐367‐Co) with different pore sizes were constructed with CoTAP and BDA/BPDA, exhibiting excellent performance of electrochemical reduction of CO_2_ to CO in water (**Figure** **7**a). By controlling the length of two linkers, the pore size and surface area of COFs are precisely controlled. Compared with COF‐366‐Co, COF‐367‐Co synthesized by the longer BPDA ligand has larger pores, which makes it higher CO_2_ adsorption capacity. At the same time, the Co‐active site shows a higher electrochemical catalytic efficiency (Figure 7b).^[^ ^117^ ^]^

Meanwhile, a unique feature of Por/Pc is that their catalytic activity strongly depends on their aggregation state. Considering this, the dimensionality of POFs is very important for catalytic performance. The application potential of bulk POFs materials to be used as electrocatalysts is largely hampered by their insufficient electrical conductivity and unavoidable gas/mass/electron diffusion barriers, which could be significantly improved by tuning the synthesis approach, especially 2D and 3D structure. For instance, the planar quadrilateral structure of Por/Pc favors the formation of 2D POF, which could expose larger surfaces and more accessible active sites, promoting the interaction with intermediate and substrate molecules. Thus, a series of strategies from the previous synthetic part (Section 2) were used to construct 2D nanosheets for electrocatalysis. Recently, a 2D Cu Por‐based MOF nanosheet (thickness about 3.7 nm) named Cu_2_(CuTCPP) MOF was constructed by Cu‐TCPP and Cu_2_(COO)_4_ nodes (Figure 7d,e).^[^ ^131^ ^]^ It showed a highly selective HCOO− (FE = 68.4%) and CH_3_COO− (FE = 16.8%) production (Figure 7f). However, the obtained 2D structure is easy to accumulate into the bulk structure, which limits the advantages of 2D structure. Compared with 2D POFs, 3D frameworks could increase more available catalytic sites, raise the current density, and reduce the possible aggregation of Por/Pc‐based building blocks. For example, the 3D Pc‐MOF with roc topology, named MOF‐1992, is an anionic framework, which is synthesized by Fe_3_(‐C_2_O_2_‐)_6_(OH_2_)_2_ trimers and tetratopic cobalt phthalocyanin‐2,3,9,10,16,17,23,24‐octaol (Figure 7g). As shown in Figure 7h,i, MOF‐1992 display high active sites with 270 nmol cm^−2^ and high CO_2_RR performance with an overpotential of about 520 mV to achieve the current density of about 16.5 mA cm−2. In addition, the 3D Por(Co/H)‐COF prepared through [4+4] condensation of TFPP‐Co and TAPM exhibited excellent CO_2_RR performance with the larger CO partial current density (*j*_CO_, 15.5 mA cm−2) than that of 2D COF‐366‐Co (13.2 mA cm−2) at 1.1 V (Figure 7j,k). Moreover, as shown in Figure 7l, the *j*_CO_ of 3D‐Por(Co/H)‐COF was also higher than that of COF‐366‐Co during the applied potential from −0.6 to −1.0 V (Figure 7l).^[^ ^36^ ^]^

#### 3.1.4 Adopting Hybrid System

Electron transfer is a very important factor in electrochemical reactions. To obtain outstanding comprehensive electrocatalytic performance, POFs can be further composited with a wide variety of conductive substrates (such as graphene, carbon nanotube (CNT), and fluorine‐doped tin oxide (FTO)) by a covalent bond or noncovalent interaction to improve the electron transfer capability.^[^ ^132^, ^133^, ^134^ ^]^ Benefiting from the larger surface area of the substrates, it can not only prevent the accumulation of POF layers but also significantly increase the density of active centers. Meanwhile, this hybrid system can improve the conductivity and the synergy between POFs and the substrate, so it effectively enhances the catalytic activity.

**Figure 8:**
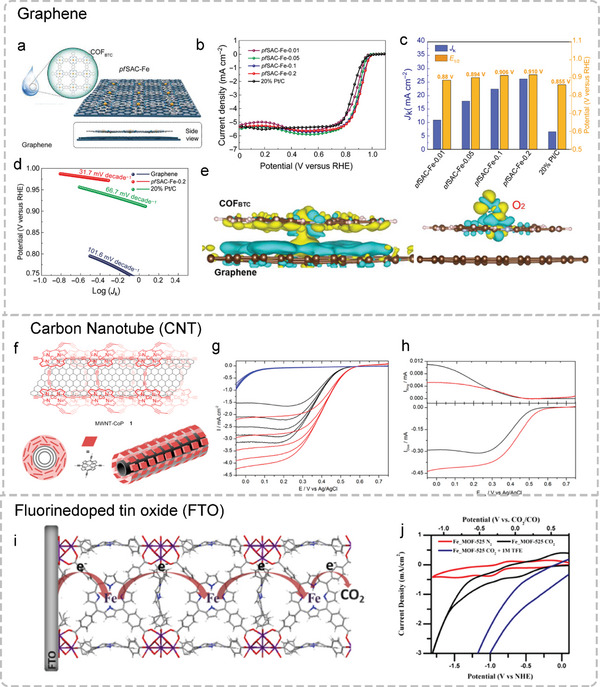
Different hybrid systems of POFs. a) Schematic diagram of the pfSAC‐Fe electrocatalyst in the synthetic process. b) LSV curves of pfSAC‐Fe‐X (X = added mass ratio of COF_BTC_) and 20% Pt/C in O_2_‐saturated 0.1 m KOH solution with the rotation speed of 1600 rpm. c) Kinetic current density (at 0.85 V vs RHE) and half‐wave potentials of different catalysts corresponding to b. d) Tafel slopes. e) Schematic diagram of differential charge density distribution on pfSAC‐Fe (left) and pfSAC‐Fe with the absorption of oxygen (right). The yellow part: Charge accumulation; the blue part: Charge decrease. Reproduced with permission.^[^ ^135^ ^]^ Copyright 2019, American Association. f) Schematic diagram of the mono‐wall carbon nanotubes coated with the POF. g) LSV curves at different rotation rates in O_2_‐saturated 0.5 m H_2_SO_4_. Red line: MWNT−CoP 1; black line: physisorbed MWNT/CoP; blue line: MWNTs. h) RRDE measurements at the rotation speed of 400 rpm. Red line: MWNT−CoP 1; black line: physisorbed MWNT/CoP; blue line: MWNTs. Reproduced with permission.^[^ ^42^ ^]^ Copyright 2014, American Chemical Society. i) Illustration of Fe‐MOF‐525 grown on FTO substrate for CO_2_RR. j) CV curves. Reproduced with permission.^[^ ^136^ ^]^ Copyright 2015, American Chemical Society.

As a 2D star material with high conductivity, graphene not only possesses a large specific surface area but also exhibit highly conjugated atomic structures as well as unique thermal and chemical stability, which can significantly promote the charge transfer between POFs and electrode. The COF containing atomically dispersed Fe‐N‐C active sites can be tightly anchored to graphene by van der Waals force to construct pfSAC‐Fe hybrids. When compared to commercial Pt/C, the obtained pfSAC‐Fe exhibit outstanding ORR performance with a higher half‐wave potential (0.91 V) and fast kinetic with the lower Tafel slopes (31.7 mV decade−1) (**Figure** **8**a–d).^[^ ^135^ ^]^ Through HAADF‐STEM analysis, the coordinated Fe atoms only existed in atomic structure and were uniformly anchored on the graphene substrate. The differential charge density distribution proves that the electrons from the carbon matrix have been gravitated to the N‐coordinated Fe active centers, constructing the Fe‐carbon electron pathway, which leads to an effective reduction in the resistivity of the material (Figure 8e). CNT is another widely used conductive carbon material to support POFs due to its high electrical conductivity and large specific surface area. A Co‐Por‐COF hybrid electrocatalyst (MWCNT‐CoP) can be constructed by covalently linking Co meso‐tetra(trimethylsilylethynyl)porphyrin (TEP) on the surface of mono‐wall CNT template (Figure 8f–h).^[^ ^42^ ^]^ The MWCNT‐CoP hybrid as the ORR electrocatalyst exhibited outstanding electrocatalytic activity in acidic media through a 4e− reaction pathway, which corresponds to the direct reduction of O_2_ to H_2_O. More importantly, due to the synergy of multiple *π*‐stacking interactions between the Por‐COF and the mono‐wall CNT as well as the abundant covalent bonds in Pc, MWCNT‐CoP displayed excellent durability with only a 5% current density decay after one‐day measurement. In addition to the common carbon materials, several studies have found that conductive FTO electrodes can also be utilized as substrates to composite with POFs. For example, an electrophoretic deposition technology was applied to construct a Fe‐Por‐MOF (Fe‐MOF‐525) grown on the FTO electrode for CO_2_RR (Figure 8i). The obtained MOF‐525‐Fe hybrid showed a high CO_2_RR performance with an overpotential of about 650 mV achieving a current density of about 4 mA mg^−1^ and a FE of 100% for CO production (Figure 8j).^[^ ^136^ ^]^ In summary, introducing a conductive substrate can increase the conductivity, catalytic activity, and even stability of POFs‐based electrocatalysts.

### 3.2 Electrocatalytic Carbon Dioxide Reduction Reaction

Converting atmospheric CO_2_ into energy‐intensive carbon compounds through electrochemical methods is promising to reduce the concentration of atmospheric CO_2_ and improve energy independence on fossil fuels.^[^ ^137^, ^138^ ^]^ Recent studies have shown that high local proton concentrations and positive charges can be achieved by modifying diverse substituents at the meso‐position of phenyl groups in Por/Pc building blocks, thus significantly boosting the CO_2_RR performance. The definite atomic structure of POFs is not only helpful in investigating the structure‐performance relationship but also exhibits efficient CO_2_RR activity due to their stable and conjugated structure as well as suitable binding affinity to CO_2_ and CO, which has a unique advantage in the study of the reaction mechanisms. Therefore, a series of POFs have been developed for CO_2_RR.^[^ ^139^ ^]^

**Figure 9:**
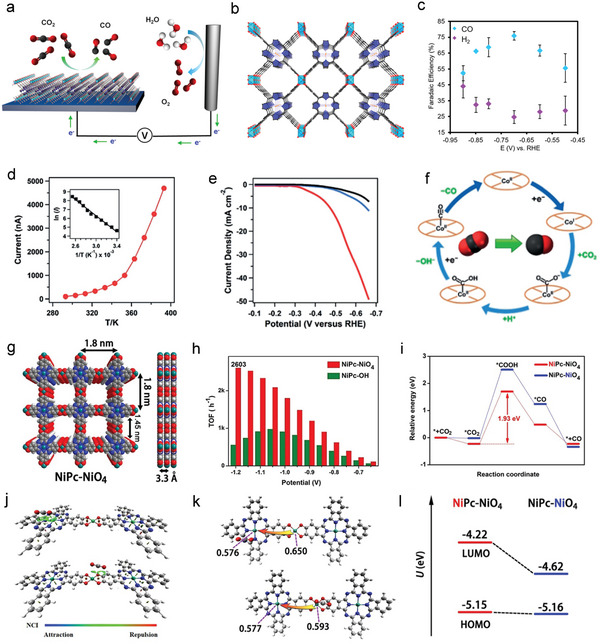
a) The Por‐MOF is integrated with a conductive substrate to build an electrochemical CO_2_RR system. b) Structure diagram of Al_2_(OH)_2_TCPP‐Co. c) FE of CO for Al_2_(OH)_2_TCPP‐Co. Reproduced with permission.^[^ ^140^ ^]^ Copyright 2015, American Chemical Society. d) Temperature‐dependent conductivity plot of CoPc‐PDQ‐COF under an applied voltage of 1 V. Inset: Arrhenius plot of the current versus temperature. e) LSV curves of CoPc‐PDQ‐COF (red) and contrast catalysts. f) The schematic diagram of the electrocatalytic CO_2_ reduction cycle by Co‐Pc. Reproduced with permission.^[^ ^41^ ^]^ Copyright 2020, Wiley‐VCH. g) Schematic diagram of NiPc‐NiO_4_. h) TOF of CO for NiPc‐NiO_4_ and NiPc‐OH. i) Calculated energy diagrams of two proposed active sites in NiPc‐NiO_4_ for CO_2_. j) The noncovalent interaction between CO_2_ and NiPc‐NiO_4_ structure. k,l) The Mulliken charge LUMO of different Ni sites in NiPc‐NiO_4_ with the introduction of CO_2_. Reproduced with permission.^[^ ^44^ ^]^ Copyright 2021, Wiley‐VCH.

As mentioned in Section 3.1, Co is the common active site of electrocatalytic CO_2_RR since the Co‐N_4_ catalytic site can remarkably reduce free energy for forming *COOH in the RDS, leading to high electroconductive activity and selectivity.^[^ ^35^ ^]^ For instance, Co‐Por as a catalytic building block was used to construct a 3D Co‐Por‐MOF catalyst toward CO_2_RR, which included first the atomic layer deposition (ALD) of metal oxide thin films as precursors of metal specials onto electrodes and subsequently the formation of Por‐MOF by reaction the ALD treated electrode with the appropriate organics in a microwave reactor (**Figure** **9**a,b).^[^ ^140^ ^]^ Remarkably, the FE and TON value of the MOFs reached 76% and 14 000, respectively (Figure 9c). In situ spectroelectrochemical measurement showed most catalytic centers were Co(I), which was reduced from Co(II) during the CO_2_RR. In addition, by designing the linkage of POFs, the electrocatalyst with high conductivity can be obtained since the linkage type will affect the crystallinity and conductivity of the material to modulate catalytic performance. Recently, one novel Pc building block (cobalt (II) 2,3,9,10,16,17,23,24‐octakis(amino) phthalocyanine, (NH_2_)_8_CoPc) has been designed to react with the linker (4,5,9,10‐pyrenediquinone, PDQ) for the construction of 2D CoPc‐PDQ‐COF.^[^ ^41^ ^]^ It is worth mentioning that the phenazine linkage offers the CoPc‐PDQ‐COF a fully conjugated structure, which provides its high stability and conductivity (Figure 9d). The FE of CoPc‐PDQ‐COF is up to 96% at −0.66 V, and the TOF value is up to 320 000, which is 32 times higher than the molecular Co‐Pc (Figure 9e). Moreover, the overall proton/electron transfer pathways of active Co‐Pc site during CO_2_RR were investigated (Figure 9f). The change from high valence state Co(II) to low valence state Co(I) occurs at the first step of the electron injection, mainly distributed on the Co *d_z_* ^2^ orbital and partly distributed on the C *p_z_* orbital of proximal carbon on the Pc ring. Then the CO2− anion was formed through a charge transfer process from Co(I) site to the coordinated CO_2_, followed by the proton transfer process that occurs during the formation of intermediate COOH*. Finally, COOH* generates CO* via proton‐electron transfer.

Instead of the Co‐N‐C catalytic center, the Ni‐N‐C‐based POFs also have been explored in the CO_2_RR process. For instance, the phenolate‐based 2D NiPc‐NiO_4_ MOF was linked by catechol‐modified Pc building block (nickel phthalocyanin‐2,3,9,10,16,17,23,24‐octaol, NiPc‐OH) and the nickel salts (Figure 9 g).^[^ ^44^ ^]^ Due to the existence of phenolate, this 2D NiPc‐NiO_4_ MOF exhibits excellent conductivity due to the high *d*‐*p* orbital overlap between catechol and nickel. The selectivity of NiPc‐NiO_4_ nanosheets for CO preparation reaches 98.4%, and the TOF of the reversible hydrogen electrode (RHE) at −1.2 V can reach 2603 h−1. Notably, this 2D NiPc‐NiO_4_ MOF displays the highest TOF and *j*_CO_ than any other known MOF‐based electrocatalysts (Figure 9 h). To further explore the active site of NiPc‐NiO_4_, the CO_2_RR reaction pathway of two different Ni active sites in the 2D MOF is calculated. As shown in Figure 9i, the energy of the formation of COOH* intermediate on the NiPc site is 1.93 eV lower than that on the NiO_4_ node (2.53 eV), suggesting that CO_2_RR may occur preferentially at the NiPc site. In terms of adsorption capacity, the van der Waals interaction between the CO_2_ molecule and NiPc is stronger than that between the NiO_4_ site (Figure 9j). The further Mulliken population analysis (Figure 9k) of the two Ni atoms suggests a more electron‐rich environment of Ni in the NiPc site than Ni in the NiO_4_ site. In addition, theoretical calculation shows that the energy level of NiPc‐NiO_4_ at the lowest unoccupied molecular orbital changes from −4.22 to −4.62 eV when the CO_2_ molecule moves from the NiPc site to the NiO_4_ site (Figure 9l), which indicates that NiPc has good reducibility. Therefore, the NiPc site is an outstanding catalytic center because of its strong CO_2_ adsorption capacity, rich electronic environment, and good reducibility. The above examples show that the electrocatalyst obtained by integrating M‐Por/Pc into frameworks has excellent performance for the electrocatalytic CO_2_ reduction reaction. Here, the CO_2_RR performances of representative POFs‐based electrocatalysts are summarized in **Table** **3**.^[^ ^36^, ^38^, ^41^, ^44^, ^76^, ^117^, ^136^, ^140^, ^141^, ^142^, ^143^ ^]^

**Table 3:** CO_2_RR performance of the representative POFs‐based electrocatalysts

CO_2_RR performance					
POFs	Main modulation strategies	Condition	Activity	Product	Ref.
Fe‐MOF‐525	Metal center; Hybrid system	1 m TBAPF_6_	FE = ≈100% TOF = 64 h^−1^	CO, H_2_	[136]
Al_2_(OH)_2_TCPP‐Co	Metal center	0.5 m KHCO_3_	FE = 76% TOF = 200 h^−1^	CO	[140]
PCN‐222 (Fe)	Metal center	0.5 m KHCO_3_	FE = 80.4% TOF = 0.012 s^−1^	CO	[141]
MOF‐1992	Metal center; Topology	0.5 m KHCO_3_	FE = 80% TOF = 0.20 s^−1^	CO	[76]
PPy@MOF‐545‐Co	Metal center; Coordination microenvironment	0.1 m KHCO_3_	FE = 98% TOF = 785 h^−1^	CO	[142]
PcCu‐O_8_‐Zn	Metal center	0.1 m KHCO_3_	FE = 88% TOF = 0.39 s^−1^	CO	[143]
NiPc‐NiO_4_	Metal center; Topology	0.5 m KHCO_3_	FE = 98.4% TOF = 2603 h^−1^	CO	[44]
COF‐366‐Co	Metal center	0.5 m KHCO_3_	FE = 90% TOF = 0.027 s^−1^	CO	[117]
COF‐367‐Co	Metal center; Topology	0.5 m KHCO_3_	FE = 91% TOF = 0.046 s^−1^	CO	[117]
COF‐366‐TTF‐Co	Metal center; Coordination microenvironment	0.5 m KHCO_3_	FE = 95% TOF = 0.188 s^−1^	CO	[38]
3D‐Por(CO/H)‐COF	Metal center; Topology	0.5 m KHCO_3_	FE = 92.4% TOF = 4610 h^−1^	CO	[36]
CoPc‐PDQ‐COF	Metal center	0.5 m KHCO_3_	FE = 91% TOF = 11 412 h^−1^	CO	[41]

### 3.3 Electrocatalytic Oxygen Reduction Reaction

ORR is one of the important reactions in a variety of clean energy storage and conversion technologies, such as fuel cells and metal‐air batteries.^[^ ^144^, ^145^, ^146^, ^147^, ^148^, ^149^, ^150^ ^]^ In the ORR process, O_2_ is first adsorbed on the surface of the catalyst. Then the O_2_ intermediate undergoes a hydrogenation reaction leading to the dissociation of the O—O bond, determining that the ORR tends to follow the 4e− or 2e− route. Finally, OH− (or H_2_O) is released into the electrolyte on the catalyst's surface.^[^ ^151^, ^152^, ^153^, ^154^ ^]^ Inspired by cytochrome P450 present in nature, which can promote the activation and reduction of O_2_, M‐N_4_‐based Por/Pc structure began to be used for electrocatalytic ORR.^[^ ^155^ ^]^ The efficiency of the ORR process mainly depends on the transport of multiple electrons from the catalyst to the oxygen molecules. M‐Por/Pc, linkers, and heteroatoms in POFs can receive and transfer electrons rapidly, which are suitable for ORR reactions with multi‐step electron transfer steps. Considerable overpotential and corresponding battery voltage loss will lead to extremely low efficiency of ORR.^[^ ^156^, ^157^ ^]^ Therefore, the development of a variety of POF‐based electrocatalysts to reduce the overpotential of the ORR process is considered to be the focus of research.

**Figure 10:**
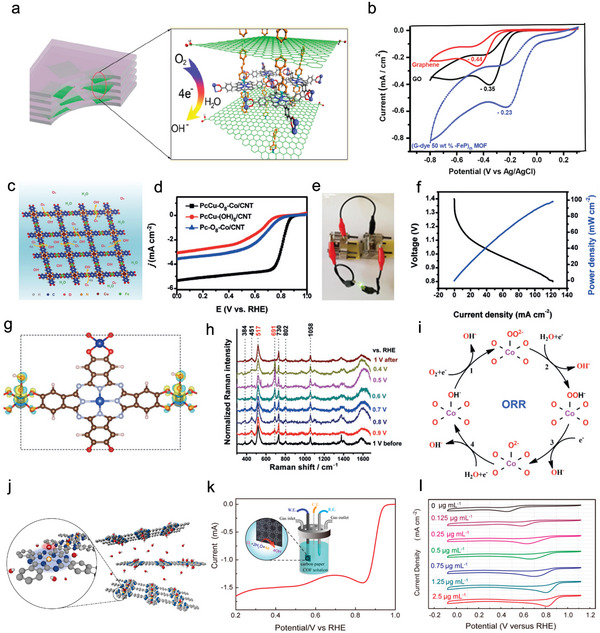
a) Schematic of (G‐dye‐FeP)*_n_* MOF. b) CV curves of different materials in 0.1 m KOH. Reproduced with permission.^[^ ^22^ ^]^ Copyright 2012, American Chemical Society. c) Schematic of PcCu‐O_8_‐Co. d) LSV curves of different catalyst in 0.1 m KOH. e) The assembled zinc–air battery device with PcCu‐O_8_‐Co/CNT and corresponding f) discharge curve and power density. g) The calculated differential charge density of PcCu‐O_8_‐Co. h) In situ Raman spectra. i) The schematic ORR reaction mechanism proposed by the derived data. Reproduced with permission.^[^ ^54^ ^]^ Copyright 2021, American Chemical Society. j) The schematic diagram of exfoliation and dissolution of COF_BTC_. k,l) LSV measurements and CV curves of the COF_BTC_ solution. Reproduced with permission.^[^ ^48^ ^]^ Copyright 2019, American Chemical Society.

Currently, doping of conductive substrate is the main strategy to improve the ORR performance of POFs. Due to the high surface areas and electroconductibility of graphene, graphene oxide (GO), and CNT, POFs can be functionalized with such carbon materials to construct highly efficient ORR catalysts. As early as 2012, a Fe‐Por‐MOF composited with pyridine‐modified rGO (py‐rGO) for ORR, namely (G‐dye‐FeP)*_n_* MOF, was developed with Fe‐TCPP building block as an organic linker and Fe3+ as node (**Figure** **10**a).^[^ ^22^ ^]^ As shown in Figure 10b, the obtained Fe‐TCPP‐MOF/py‐G hybrid exhibited an onset of 0.93 V (vs RHE) in 0.1 m KOH. The (G‐dye‐FeP)*_n_* MOF showed high ORR activity since the functionalized pyridine groups interacted with the metal sites of Por.

Additionally, CNTs are another kind of common conductive material because of their excellent conductivity. The 2D conjugated‐MOF (namely PcCu‐O_8_‐Co) with layered stacking structures can be constructed by the square‐planar linkages (cobalt bis(dihydroxy) complexes), which further combined with conductive CNTs to obtain hybrid materials (PcCu‐O_8_‐Co/CNT) (Figure 10c).^[^ ^54^ ^]^ The ORR performance results showed that PcCu‐O_8_‐Co/CNT exhibited superior activity with an E_1/2_ = 0.83 V (vs RHE) for ORR in 0.1 m KOH, outperforming other reported intrinsic MOF‐based catalysts because of its porous structure, high conductivity, and high coverage of electrocatalytic Co sites (Figure 10d). Benefiting from the excellent electrocatalytic activity of PcCuO_8_‐Co/CNT in alkaline solution, PcCuO_8_‐Co/CNT can be further applied as the air electrode catalyst for the application of zinc–air batteries primarily. The POF‐based electrocatalyst assembled zinc–air battery device exhibited outstanding performance with a high discharge current density of 120 mA cm−2 at 0.8 V, and a maximum power density of 94 mW cm−2, which are even superior to those of commercial Pt/C assembled devices (95.5 mA cm−2 and 76.4 mW cm−2, respectively (Figure 10e,f). Remarkably, such heterometal–organic frameworks have been utilized to explore the ORR mechanism further comprehensively. The DFT calculation showed Co site showed higher adsorption energy for intermediate OOH− compared with Cu‐N_4_, which could act as the active center in the ORR process (Figure 10 g). Meanwhile, in situ Raman spectra have been utilized to monitor the electrocatalytic reaction process, which first observed the adsorption peak of OH species on the catalytic CoII site to form Co^II^‐OH intermediate (517 cm−1) (Figure 10 h). During the ORR process, the Co^II^‐O peak gradually decreased with the rising voltage due to the conversion from Co^II^ to Co^III^ species, and the intensity of the Co^III^‐O oxygen intermediate (691 cm−1) increased accordingly. Overall, the combination of the theoretical and experimental results suggests that the unique Co‐O site in the PcCu‐O_8_‐Co plays a key role and acts as the active center during the ORR process. A proposed ORR mechanism in Figure 10i exhibits the ORR reaction circle based on the Co‐O site.

Besides Por/Pc‐based MOF materials, Por/Pc‐based COFs may swell in aqueous solutions due to their cross‐linking properties, which limits their processing performance and applications. To meet this challenge, the stable COF aqueous solution has been investigated. As shown in Figure 10j, the Fe‐Pc‐COF (COF_BTC_) has been prepared from the building blocks of benzene‐1,2,4,5‐tetracarbonitrile (BTC), exhibiting the rich electrons in the plane direction and high solubility after in situ exfoliation.^[^ ^48^ ^]^ In an alkaline solution, the hydroxyl group can adsorb on the positively charged Fe center, thus OH− will create a strong interaction with the COF_BTC_ layer to realize the in situ exfoliation of COF and prevent the aggregation and deposition of the stripped layer from forming a highly stable solution system. The X‐ray absorption near edge structure spectroscopic measurements of Fe in COF_BTC_ solution show that the centrosymmetric Fe‐N_4_ structure is the active site. The COF_BTC_ with abundant Fe‐N‐C sites and high conjugated structure shows outstanding catalytic activity in ORR electrocatalysis (E_1/2_ = 0.90 V) (Figure 10k). As shown in Figure 10l, the ORR performance of COF_BTC_ suggests a direct linear relationship with the catalyst concentration. Notably, the obtained COF_BTC_ solution is promising to be a substitute catalyst for Pt in zinc–air batteries. Unlike conventional catalyst‐coated carbon paper/gas diffusion layer electrodes as the air cathode, the COF_BTC_ solution can be directly mixed with the electrolyte and used as the ORR catalyst. Completely dissolving the catalyst in the electrolyte not only greatly simplifies the electrode assembly process but also improves the utilization of the catalyst, so that its electrocatalytic activity will be more stable. Benefiting from the highly exposed active sites in a homogenous solution, the COF_BTC_ solution catalyst is superior to the conventional flow cell; the catalyst needs to be deposited on the electrode surface.

### 3.4 Electrocatalytic Water‐Splitting (Hydrogen and Oxygen Evolution Reaction)

The electrocatalytic water‐splitting reaction contains the hydrogen evolution reaction and oxygen evolution reaction, which has attracted much more attention as sustainable clean energy of hydrogen in the event of a supply crisis.^[^ ^158^ ^]^ Compared to the more studied multi‐electron reactions such as CO_2_RR and ORR, the application of POF‐based materials in electrocatalytic water‐splitting is still less reported. Here, several POF‐based water‐splitting electrocatalysts will be discussed.

**Figure 11:**
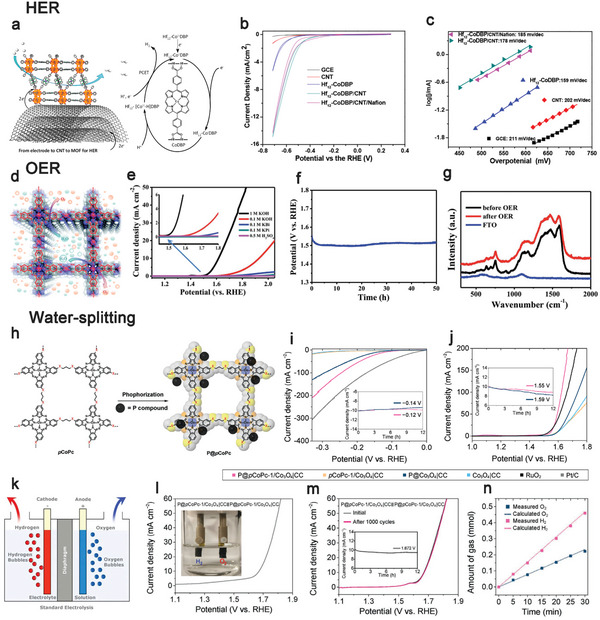
a) Schematic diagram of HER electrocatalytic process of Hf_12_−CoDBP/CNT. b) CV curves of different catalysts. c) Tafel slope of different materials. Reproduced with permission.^[^ ^160^ ^]^ Copyright 2018, American Chemical Society. d) Schematic diagram of the preparation of NiPc‐MOF. e) pH‐dependent LSV curves in different electrolytes using the NiPc–MOF as the OER catalyst. f) The long‐term chronopotentiometry measurement for OER at a constant current density of 1.0 mA cm−2. g) Raman spectra of different catalysts. Reproduced with permission.^[^ ^163^ ^]^ Copyright 2018, The Royal Society of Chemistry. h) Schematic diagram of *p*CoPc and P@*p*CoPc. LSV curves in 1 m KOH for i) HER and j) OER. k) Schematic diagram of the water decomposition process. LSV curves of P@pCoPc‐1/Co_3_O_4_|CC l) in overall water splitting and m) the comparison before and after 1000 cycles. Insets in (l) and (m) are the setup for overall water splitting and the long‐term chronoamperometric measurement at a constant potential of 1.672 V for 12 h, respectively. n) The experimental and calculated amounts of H_2_ and O_2_ as a function of time during the overall water splitting at a constant current density of 50 mA cm−2. Reproduced with permission.^[^ ^43^ ^]^ Copyright 2021, Wiley‐VCH.

HER is a reaction process that involves two‐electron transfer. In an acidic solution, H+ is first adsorbed on the initial active site to generate H*. Then, through Tafel or Heyrovsky path, H_2_ can be obtained. Generally, the reactions of H_2_‐producing are inhibited when the binding of H+ to the catalyst surface is weak.^[^ ^159^ ^]^ POF‐based materials can provide low overpotential and high catalytic activity for the HER electrocatalytic process. For instance, a Hf_12_‐Por‐based MOF can be loaded with CNTs through covalent adsorption to improve the conductivity, which will facilitate the electrons transfer from the electrode to M‐Por active sites, resulting in outstanding HER performance (**Figure** **11**a). The thickness of MOF on the AFM image surface of Hf_12_‐CoDBP shows only 20–25 nm. The covalent adsorption of Hf_12_‐CoDBP/CNT does not change the morphology of MOF itself and shows a high electrocatalytic effect at different potentials. The obtained Hf_12_‐CoDBP/CNT hybrid displayed an outstanding electrocatalytic HER performance in the acidic media, which only acquired 650 mV overpotential to achieve the current density of 10 mA cm−2 (Figure 11b). The calculated Tafel slope of the optimal catalyst is 178 mV dec−1, suggesting the RDS belongs to the Volmer step, which depends on the one proton adsorption on the catalytic site (Figure 11c).^[^ ^160^ ^]^

OER, which involves the four‐electron transfer process, is generally considered as the bottleneck for effective hydrogen evolution by splitting water.^[^ ^161^, ^162^ ^]^ In an alkaline solution, OH− is first adsorbed on the initial active site to generate *OH. After the protons and electrons of *OH are removed, *O intermediates are generated. Subsequently, two kinds of *O can be combined to form O_2_, or through a two‐step and proton‐coupled electron transfer process, *OOH can be obtained first, and O_2_ can be finally obtained. The overall oxidation process of water at least asks for the endothermic energy of 237 kJ mol−1, and the atom rearrangement always occurs on the catalyst surface, which leads to the slow kinetics of the OER process.^[^ ^35^ ^]^ POFs, as specific kinds of framework material with clear atomic structure and adjustable coordination environment, are powerful for studying the OER mechanism and building the structure‐performance relationship. For instance, the 2D NiPc‐MOF materials were constructed using a Pc‐based building unit (2,3,9,10,16,17,23,24‐octaamino‐phthalocyaninato nickel, NiPc‐NH_2_) for OER electrocatalysis (Figure 11d).^[^ ^163^ ^]^ As shown in Figure 11e, the electrocatalytic activity of NiPc‐MOF in the OER process increased with the pH value. Notably, the NiPc‐MOF showed excellent OER activity in 1 m KOH, which only acquires 250 mV overpotential to achieve 10 mA cm−2. Notably, the calculated Tafel slope of NiPc‐MOF is 74 mV decade−1, indicating the fast reaction kinetics. The long‐term stability tests of NiPc–MOF observe that the catalytic activity only slightly declined after 50 h of working time (Figure 11f). Subsequently, the Raman spectrum (Figure 11 g) of the NiPc‐MOF before and after the OER measurement shows that the main structure of NiPc‐MOF remains the same.

Due to the promising HER and OER activity of POFs‐based electrocatalysts that can overcome the shortcomings of precious metal HER/OER electrocatalysts of scarcity and high cost, they can be further applied for the water splitting reaction, including both HER and OER process. Massive works have been devoted to developing efficient POFs‐based bifunctional electrocatalysts for simultaneously facilitating the OER and HER process. Among them, heteroatom doping, like S and P, is an effective method to improve the electrocatalytic activities of POFs‐based electrocatalysts and promote bifunctional catalytic activity. For example, two new water‐splitting catalysts with adjustable electrochemical performance were constructed, which were named *p*CoPc‐1 and *p*CoPc‐2, containing S linkers and SO_2_ linkers, respectively (Figure 11 h).^[^ ^43^ ^]^ The *p*CoPc‐1 and *p*CoPc‐2 showed excellent bifunctional electrocatalytic activities toward HER and OER. In order to further improve the catalytic performance, the phosphorylation process has been applied for *p*CoPc to form P@*p*CoPc to boost its hydrophilicity and conductivity, thus acting as a proton acceptor to accelerate the HER catalytic reaction. Then, the bifunctional electrocatalytic activity of *p*CoPcs and P@*p*CoPcs were systematically investigated, which were all coated on a variety of substrates, such as SiO_2_, conductive carbon cloth (CC), and CoO‐ or Co_3_O_4_‐modified CC. Particularly, the P@*p*CoPc‐1/Co_3_O_4_|CC exhibited the best bifunctional electrocatalytic performance toward HER and OER. As shown in Figure 11i,j, the overpotentials of P@*p*CoPc‐1/Co_3_O_4_|CC about OER and HER performance are as low as 320 and 120 mV at the current density of 10 mA cm−2, respectively.

Inspired by the excellent bifunctional electrocatalytic activity of P@pCoPc‐1/Co_3_O_4_|CC, a two‐electrode cell has been assembled by the catalyst as both anode and cathode for evaluating its water‐splitting capacity (Figure 11k). Notably, the assembled P@pCoPc‐1/Co_3_O_4_|CC device requires a small overpotential of 1.672 V to achieve 10 mA cm−2. The long‐term stability test suggests no obvious decline of electrocatalytic performance after continuous 12 h working time and 1000 cycles operation (Figure 11l,m). To explore the FE of the water splitting by P@pCoPc‐1/Co_3_O_4_|CC, the volumes of H_2_ and O_2_ evolution have been recorded to compare with the calculated volumes. The experimental and calculated amounts of H_2_ and O_2_ at a current density of 50 mA cm−2 for 30 min are presented in Figure 11n. The ratio between the amount of H_2_ and O_2_ generated is 2.06, which is close to the theoretical value. This work shows the multifunctional tunability of POF‐based materials for electrocatalysis, which shows the promising application in water splitting in the future.

## 4 Conclusions and Future Perspectives

**Figure 12:**
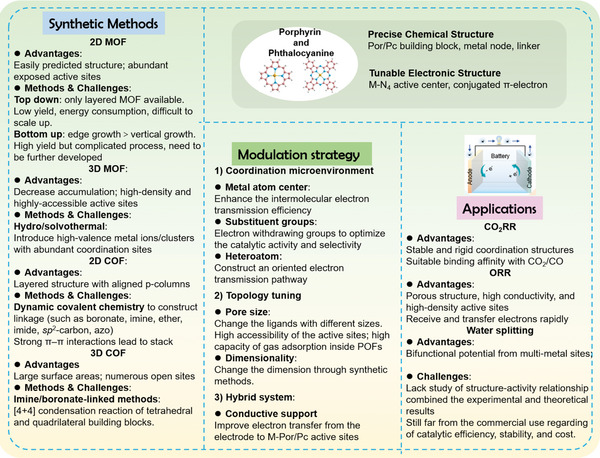
The summary of synthetic methods, modulation strategy, and electrocatalytic applications of POFs.

In summary, we have reviewed recent achievements and given comments on current advances in designing POFs for efficient electrocatalytic applications in this new progress report (**Figure** **12**). Benefiting from their unique metal‐N_4_ coordination structure, high conjugated *π*‐electron system, flexibly tunable components, and chemical stability, various POFs‐based catalysis have been constructed for efficient electrocatalytic applications. This review details numerous synthesis methods of POFs, and describes the advantages and disadvantages of these strategies. At the same time, the booming developments and the specific ways for modulating their chemical and electronic structures, including tailoring coordination microenvironment (metal atom center, substituent groups, and heteroatom), modulation of topology (pore size and dimensionality), and hybrid system, are also fully reviewed and emphasized.

In short, representative research advances of POFs‐based catalysts in electrocatalysis are summarized. Although the investigations on the electrocatalytic field of POFs‐based catalysts are still in their infancy, they have aroused significant appeal. In addition, except for these potential investigations, comprehensive evaluation of the detailed structural characterization, including chemical structures, electronic properties, complex compositions, coordination microenvironments, and hybrid substrates, is still challenging. 1) For the current synthetic strategies for POFs, the universality and scalability of them are still needed to improve. The limited reaction types, chemical structures, and too many synthesis steps of POFs are a hindrance to large‐scale industrial production. Thus, developing a facilitated, green, low‐cost, and large‐scale method for commercial electrocatalysts is urgent. In summary, there will be a huge market prospect for POFs‐based electrocatalysts if the above disadvantages about commercial production of them will be solved in the future. To promote the industrial production of POFs, we should optimize existing synthetic strategies and develop new approaches in the future. Additionally, it is necessary to create more POFs configurations to develop its inherent advantages in electrochemical catalysis. Meanwhile, theoretical calculations as guidance could be used to develop new large‐scale synthesis methods and guide to constructing of novel POFs‐based electrocatalysts.

2) So far, POFs have been constructed to combine many metal ions into their M‐N_4_ sites, such as Fe, Co, Ni Cu, and Zn, but there is great hope that other metal elements can be combined with POFs to expand further the electrocatalyst class, including main group elements, other transition metal elements, and lanthanides. Developing unique POFs‐based materials with diverse chemical compositions, coordination environments, and basic physical properties will inspire more electrocatalysts based on POFs toward high performance.

3) Although the catalytic activity of POFs can be optimized by coordination microenvironment regulation strategies, such as functionalization of substituent groups or doping heteroatom, it is challenging to precisely control the chemical structure of POFs‐based materials. For example, the introduction of electron‐withdrawing groups or second atoms usually increases the difficulty of synthesis. Meanwhile, the electronic structure and mechanism are difficult to explore due to their uncontrollably precise chemical structures. Besides, the usual modulation strategies may result in side effects such as instability, poor conductivity, and even impurity. Thus, it should be considered to develop the precise structure of POFs focusing on an atomic level in the future and take all factors into consideration.

4) The topology modulation strategy can significantly increase electroconductivity, boost the accessibility of the active sites, and improve the catalytic performance of POFs‐based electrocatalysts. However, the relationship between structure and activity as well as preparation conditions, are crucial points for successful topology modulation. For example, i) the ultrathin nanostructure of 2D POFs can improve the conductivity and exposed active sites, while the layer stacking caused by the strong *π*‐*π* interaction should be avoidable; ii) the interpenetration is easy to appear in 3D POFs containing large pore size, which will seriously reduce the porosity and affects the mass transfer. iii) the experimental conditions need to be strictly controlled to realize the controllable synthesis of the 2D/3D structures. Additionally, the structure evolution and topology selection can be guided by advanced in situ characterization and theoretical calculation techniques, respectively.

5) Electrical conductivity is a critical factor in the rapid transfer of electrons in electrocatalytic reactions. The conductivity of POFs is still limited, and they need to be loaded on a conductive substrate when applied in the electrochemical field. However, such a hybrid system exists the problem of phase separation and shedding of the catalyst during the catalytic process. Therefore, exploring and innovating the modified substrates to load POFs through strong interaction can be a good choice.^[^ ^8^ ^]^ In addition, to find the novel substrate, synthesizing highly conductive 2D POFs is another promising direction.

6) In acidic, alkaline, high temperature, and other harsh conditions, POFs will likely undergo structural decomposition or chemical composition change in the catalytic process. The thermal and chemical stability of Por/Pc‐MOFs can be improved by employing high oxidation state metal central clusters, such as Zr4+.^[^ ^35^ ^]^ For example, zirconium‐based porphyrin MOFs are usually extremely stable in strong acid/base solutions.^[^ ^73^ ^]^ In contrast, COF has higher stability due to covalent bonding. How to study the recognized principle of COF crystallization for improving the crystallinity of COF materials and increasing the stability of materials is one of the key issues in the following research.

7) Apart from the regulation methods on the structure or topology of POFs at a molecular level, engineering nano morphology of POFs is also one of the effective approaches to improve the electrocatalytic performance further. Classified by the specific geometrical shape and size rather than the specific molecular dimensions, the common nano morphologies of POFs are concluded as 0D (nanoparticles) with high stability and dispersion, 1D (nanoribbons and fibers) with high phase purity and fewer defects, 2D (films and membranes) owning access to catalytic interfaces, and 3D (hierarchical nanostructures and aerogels) combined multi‐advantages from low‐dimensional nanostructure.^[^ ^164^ ^]^ Those specific morphologies of POFs provide them with unique properties more suitable for electrochemical applications. In the future, research on nano‐morphological evolution and corresponding applications should be concerned.

8) So far, due to diverse intermediates and products, uncertain reaction paths, and complex multiple electron transfers of electrocatalytic applications, including CO_2_RR, ORR, HER, and OER, it is challengeable to determine the catalytic mechanism and select efficient electric catalysts. The comprehensive study of the catalytic mechanism of POFs is conducive to the reasonable structural design and electrocatalytic materials with better catalytic activity. Therefore, a variety of progressive in situ techniques, including XRD, X‐ray photoelectron spectroscopy, X‐ray absorption fine structure spectroscopy (XAS), Raman spectroscopy, Fourier transform infrared spectroscopy, TEM and other advanced in situ techniques are urgently needed for in‐depth characterization and analysis of the relationship between the chemical structures and electrocatalytic activities of materials. **Table** **4** presents a comprehensive summary of the advantages, disadvantages, and key issues of the above standard in situ techniques.^[^ ^165^, ^166^, ^167^, ^168^, ^169^, ^170^ ^]^

**Table 4:** Summary of the advantages, disadvantages, and key issues identified from each in situ technique

In situ techniques	Advantages	Disadvantages	Key issues identified	Ref.
XRD	Tracking the structural evolution of POFs‐based catalysts from the degree of crystallinity and phase structure type.	Unapplicable to electrocatalysts with amorphous structure or low crystallinity.	(1) Catalyst stability.	[165]
XPS	Probing the surface structural evolution information before and after the electrochemical reaction.	Hardly capture the real catalyst surface structure because of the inescapable contact with air in the process of detection.	(1) Active sites, (2) Catalyst stability.	[166]
XAS	Investigating the valence state and coordination structure of catalytic sites POFs‐based catalysts.	Hardly show the information on catalytic reactions occurring at the catalyst surface.	(1) Key intermediates, (2) Reaction mechanism, (3) Active sites, (4) Catalyst stability.	[167]
FTIR	Detecting adsorbed species on the surface of POFs‐based catalysts.	Hardly distinguish the spectra from electrolytes and catalysts’ interfaces due to the interference from other components.	(1) Key intermediates, (2) Reaction mechanism, (3) Reaction environment.	[168]
Raman	Identifying the key intermediate species and the catalytic sites of POFs‐based catalysts.	The corresponding reaction intermediates hardly be detected due to the corresponding weak Raman signals.	(1) Key intermediates, (2) Reaction mechanism, (3) Active sites, (4) Reaction environment.	[169]
TEM	Observing the structural change of POFs‐based catalysts.	The fabrication of the in situ cell and the electron beam damage.	(1) Catalyst stability.	[170]

In conclusion, as a novel research direction in electrocatalysis (CO_2_RR, ORR, and water splitting), POFs‐based materials have attracted much attention. A series of synthetic methods and modification strategies are used to construct POFs‐based materials with high tunability and catalytic activities for electrocatalytic applications. POFs‐based electrocatalysts with high catalytic activity and high tunability can be obtained through various synthetic methods and modification strategies. Modulating the chemical structures, coordination microenvironment, topologies, and hybrid substrates of the POFs‐based materials has been proven an effective way to improve their activity. To promote the possibility for commercialization of POFs‐based materials, the design strategies and potential mechanisms of POFs‐based electrocatalysts should be further investigated. We firmly believe that the research progress in the synthesis‐structure‐performance relationship of POFs will considerably encourage their wide range of applications for sustainability, energy, and electrocatalysis. We believe that the demonstration of the synthesis‐structure‐performance correlations on POFs and their guided discovery will considerably encourage their wide range of applications for sustainability, energy, and electrocatalysis. Overall, this comprehensive review focusing on the frontier will provide multidisciplinary and multi‐perspective guidance for the subsequent experimental and theoretical progress of POFs and reveal their key challenges and application prospects in future electrocatalytic energy conversion systems.

## Conflict of Interest

The authors declare no conflict of interest.

## References

[1] N. Cox , M. Retegan , F. Neese , D. A. Pantazis , A. Boussac , W. Lubitz , Science 2014, 345, 804. 25124437 10.1126/science.1254910

[2] G. P. Peters , R. M. Andrew , J. G. Canadell , P. Friedlingstein , R. B. Jackson , J. I. Korsbakken , C. Le Quere , A. Peregon , Nat. Clim. Change 2020, 10, 3.

[3] E. Pomerantseva , F. Bonaccorso , X. Feng , Y. Cui , Y. Gogotsi , Science 2019, 366, 969. 10.1126/science.aan8285 31753970

[4] Y. Sun , N. Liu , Y. Cui , Nat. Energy 2016, 1, 16071.

[5] M. G. Walter , E. L. Warren , J. R. McKone , S. W. Boettcher , Q. X. Mi , E. A. Santori , N. S. Lewis , Chem. Rev. 2010, 110, 6446. 21062097 10.1021/cr1002326

[6] Y. Lee , J. Suntivich , K. J. May , E. E. Perry , Y. Shao‐Horn , J. Phys. Chem. Lett. 2012, 3, 399. 26285858 10.1021/jz2016507

[7] T. Hisatomi , J. Kubota , K. Domen , Chem. Soc. Rev. 2014, 43, 7520. 24413305 10.1039/c3cs60378d

[8] M. Lu , M. Zhang , J. Liu , Y. Chen , J. P. Liao , M. Y. Yang , Y. P. Cai , S. L. Li , Y. Q. Lan , Angew. Chem., Int. Ed. 2022, 61, 202200003. 10.1002/anie.202200003 35060268

[9] C. Gao , S. Mu , R. Yan , F. Chen , T. Ma , S. Cao , S. Li , L. Ma , Y. Wang , C. Cheng , Small 2022, 18, 2105409. 10.1002/smll.202105409 35023628

[10] Z. Zhang , W. Wang , X. Wang , L. Zhang , C. Cheng , X. Liu , Chem. Eng. J. 2022, 435, 133872.

[11] T. Wang , W. Wang , W. Shao , M. Bai , M. Zhou , S. Li , T. Ma , L. Ma , C. Cheng , X. Liu , ChemSusChem 2021, 14, 5112. 34520128 10.1002/cssc.202101844

[12] B. Zhang , Y. Zheng , T. Ma , C. Yang , Y. Peng , Z. Zhou , M. Zhou , S. Li , Y. Wang , C. Cheng , Adv. Mater. 2021, 33, 2006042. 10.1002/adma.202006042 PMC11468660 33749910

[13] G. Wu , P. Zelenay , Acc. Chem. Res. 2013, 46, 1878. 23815084 10.1021/ar400011z

[14] H.‐x. Zhong , J. Wang , Y.‐w. Zhang , W.‐l. Xu , W. Xing , D. Xu , Y.‐f. Zhang , X.‐b. Zhang , Angew. Chem., Int. Ed. 2014, 53, 14235. 10.1002/anie.201408990 25331053

[15] Z. Xu , X. Zhuang , C. Yang , J. Cao , Z. Yao , Y. Tang , J. Jiang , D. Wu , X. Feng , Adv. Mater. 2016, 28, 1981. 26753773 10.1002/adma.201505131

[16] L. M. Dai , Acc. Chem. Res. 2013, 46, 31. 23030244 10.1021/ar300122m

[17] Z. W. Chen , D. Higgins , A. P. Yu , L. Zhang , J. J. Zhang , Energy Environ. 2011, 4, 3167.

[18] F. Meng , H. Zhong , D. Bao , J. Yan , X. Zhang , J. Am. Chem. Soc. 2016, 138, 10226. 27463122 10.1021/jacs.6b05046

[19] J. Liang , R. F. Zhou , X. M. Chen , Y. H. Tang , S. Z. Qiao , Adv. Mater. 2014, 26, 6074. 25042569 10.1002/adma.201401848

[20] J. Masa , W. Xia , M. Muhler , W. Schuhmann , Angew. Chem., Int. Ed. 2015, 54, 10102. 10.1002/anie.201500569 26136398

[21] D. Guo , R. Shibuya , C. Akiba , S. Saji , T. Kondo , J. Nakamura , Science 2016, 351, 361. 26798009 10.1126/science.aad0832

[22] M. Jahan , Q. L. Bao , K. P. Loh , J. Am. Chem. Soc. 2012, 134, 6707. 22439970 10.1021/ja211433h

[23] C. T. Carver , B. D. Matson , J. M. Mayer , J. Am. Chem. Soc. 2012, 134, 5444. 22394189 10.1021/ja211987f

[24] S. Liu , Z. Wang , S. Zhou , F. Yu , M. Yu , C.‐Y. Chiang , W. Zhou , J. Zhao , J. Qiu , Adv. Mater. 2017, 29, 1700874. 10.1002/adma.201700874 28627127

[25] C. X. Zhao , B. Q. Li , J. N. Liu , Q. Zhang , Angew. Chem., Int. Ed. 2021, 60, 4448. 10.1002/anie.202003917 32315106

[26] T. M. Tang , Z. L. Wang , J. Q. Guan , Adv. Funct. Mater. 2022, 32, 2111504.

[27] G. T. Babcock , Proc. Natl. Acad. Sci. USA 1999, 96, 12971.10557256

[28] T. Kitagawa , J. Inorg. Biochem. 2000, 82, 9. 11132644 10.1016/s0162-0134(00)00155-0

[29] A. Rezaeifard , M. Jafarpour , Catal. Sci. Technol. 2014, 4, 1960.

[30] J. S. M. Lee , A. I. Cooper , Chem. Rev. 2020, 120, 2171. 31990527 10.1021/acs.chemrev.9b00399 PMC7145355

[31] I. M. Honicke , I. Senkovska , V. Bon , I. A. Baburin , N. Bonisch , S. Raschke , J. D. Evans , S. Kaskel , Angew. Chem., Int. Ed. 2018, 57, 13780. 10.1002/anie.201808240 30160076

[32] C. J. Doonan , D. J. Tranchemontagne , T. G. Glover , J. R. Hunt , O. M. Yaghi , Nat. Chem. 2010, 2, 235. 21124483 10.1038/nchem.548

[33] E. Klontzas , E. Tylianakis , G. E. Froudakis , Nano Lett. 2010, 10, 452. 20050693 10.1021/nl903068a

[34] L. Feng , K. Y. Wang , E. Joseph , H. C. Zhou , Trends Chem. 2020, 2, 555.

[35] Z. Z. Liang , H. Y. Wang , H. Q. Zheng , W. Zhang , R. Cao , Chem. Soc. Rev. 2021, 50, 2540. 33475099 10.1039/d0cs01482f

[36] S.‐Y. Chi , Q. Chen , S.‐S. Zhao , D.‐H. Si , Q.‐J. Wu , Y.‐B. Huang , R. Cao , J. Mater. Chem. A 2022, 10, 4653.

[37] B. Han , Y. Jin , B. Chen , W. Zhou , B. Yu , C. Wei , H. Wang , K. Wang , Y. Chen , B. Chen , J. Jiang , Angew. Chem., Int. Ed. 2022, 61, 202114244. 10.1002/anie.202114244 34716743

[38] H. J. Zhu , M. Lu , Y. R. Wang , S. J. Yao , M. Zhang , Y. H. Kan , J. Liu , Y. Chen , S. L. Li , Y. Q. Lan , Nat. Commun. 2020, 11, 497. 31980641 10.1038/s41467-019-14237-4 PMC6981265

[39] M. Lu , M. Zhang , C. G. Liu , J. Liu , L. J. Shang , M. Wang , J. N. Chang , S. L. Li , Y. Q. Lan , Angew. Chem., Int. Ed. 2021, 60, 4864. 10.1002/anie.202011722 33179405

[40] X. M. Yu , Y. C. Ma , C. Y. Li , X. Y. Guan , Q. R. Fang , S. L. Qiu , Chem. Res. Chin. Univ. 2022, 38, 167.

[41] N. Huang , K. H. Lee , Y. Yue , X. Xu , S. Irle , Q. Jiang , D. Jiang , Angew. Chem., Int. Ed. 2020, 59, 16587. 10.1002/anie.202005274 32436331

[42] I. Hijazi , T. Bourgeteau , R. Cornut , A. Morozan , A. Filoramo , J. Leroy , V. Derycke , B. Jousselme , S. Campidelli , J. Am. Chem. Soc. 2014, 136, 6348. 24717022 10.1021/ja500984k

[43] Y. Kim , D. Kim , J. Lee , L. Y. S. Lee , D. K. P. Ng , Adv. Funct. Mater. 2021, 31, 2103290.

[44] T. Liang , L. Zeng , Y. Shi , H. Pan , P. K. Chu , K. W. K. Yeung , Y. Zhao , Bioact. Mater. 2021, 6, 3049. 33778187 10.1016/j.bioactmat.2021.02.025 PMC7960947

[45] J. Park , Q. Jiang , D. Feng , L. Mao , H. C. Zhou , J. Am. Chem. Soc. 2016, 138, 3518. 26894555 10.1021/jacs.6b00007

[46] E. X. Chen , M. Qiu , Y. F. Zhang , L. He , Y. Y. Sun , H. L. Zheng , X. Wu , J. Zhang , Q. Lin , Angew. Chem., Int. Ed. 2022, 61, 202111622. 10.1002/anie.202111622 34652055

[47] X. Wang , M. Bahri , Z. Fu , M. A. Little , L. Liu , H. Niu , N. D. Browning , S. Y. Chong , L. Chen , J. W. Ward , A. I. Cooper , J. Am. Chem. Soc. 2021, 143, 15011. 34516737 10.1021/jacs.1c08351

[48] P. Peng , L. Shi , F. Huo , S. Zhang , C. Mi , Y. Cheng , Z. Xiang , ACS Nano 2019, 13, 878. 30609343 10.1021/acsnano.8b08667

[49] Y. Zhang , J. F. Lovell , Wiley Interdiscip. Rev.: Nanomed. Nanobiotechnol. 2017, 9, 1420. 10.1002/wnan.1420 PMC5179311 27439671

[50] J. P. Collman , R. Boulatov , C. J. Sunderland , L. Fu , Chem. Rev. 2004, 104, 561. 14871135 10.1021/cr0206059

[51] E. I. Solomon , D. E. Heppner , E. M. Johnston , J. W. Ginsbach , J. Cirera , M. Qayyum , M. T. Kieber‐Emmons , C. H. Kjaergaard , R. G. Hadt , L. Tian , Chem. Rev. 2014, 114, 3659. 24588098 10.1021/cr400327t PMC4040215

[52] S. A. Younis , D. K. Lim , K. H. Kim , A. Deep , Adv. Colloid Interface Sci. 2020, 277, 102108. 32028075 10.1016/j.cis.2020.102108

[53] J. Mack , Chem. Rev. 2017, 117, 3444. 28222605 10.1021/acs.chemrev.6b00568

[54] H. X. Zhong , K. H. Ly , M. C. Wang , Y. Krupskaya , X. C. Han , J. C. Zhang , J. Zhang , V. Kataev , B. Buchner , I. M. Weidinger , S. Kaskel , P. Liu , M. W. Chen , R. H. Dong , X. L. Feng , Angew. Chem., Int. Ed. 2019, 58, 10677. 10.1002/anie.201907002 31169942

[55] S. De , T. Devic , A. Fateeva , Dalton Trans. 2021, 50, 1166. 33427825 10.1039/d0dt03903a

[56] J. Chen , Y. Zhu , S. Kaskel , Angew. Chem., Int. Ed. 2021, 60, 5010. 10.1002/anie.201909880 PMC7984248 31989749

[57] X. Wu , S. Xiao , Y. Long , T. Ma , W. Shao , S. Cao , X. Xiang , L. Ma , L. Qiu , C. Cheng , C. Zhao , Small 2022, 18, 2105831. 10.1002/smll.202105831 35102688

[58] F. Chen , Q. Tang , T. Ma , B. Zhu , L. Wang , C. He , X. Luo , S. Cao , L. Ma , C. Cheng , Infomat 2022, 4, e12299.

[59] M. Velicky , P. S. Toth , Appl. Mater. Today 2017, 8, 68.

[60] H. Zhang , Chem. Rev. 2018, 118, 6089. 29991267 10.1021/acs.chemrev.8b00278

[61] S. Khan , M. Shahid , Mater. Adv. 2021, 2, 4914.

[62] S. Wu , L. Qin , K. Zhang , Z. Xin , S. Zhao , RSC Adv. 2019, 9, 9386. 35520711 10.1039/c9ra00662a PMC9062198

[63] Q. Y. Jiang , C. H. Zhou , H. B. Meng , Y. Han , X. F. Shi , C. H. Zhan , R. F. Zhang , J. Mater. Chem. A 2020, 8, 15271.

[64] M. C. Wang , H. H. Shi , P. P. Zhang , Z. Q. Liao , M. Wang , H. X. Zhong , F. Schwotzer , A. S. Nia , E. Zschech , S. Q. Zhou , S. Kaskel , R. H. Dong , X. L. Feng , Adv. Funct. Mater. 2020, 30, 2002664.

[65] Y. Ding , Y. P. Chen , X. Zhang , L. Chen , Z. Dong , H. L. Jiang , H. Xu , H. C. Zhou , J. Am. Chem. Soc. 2017, 139, 9136. 28651432 10.1021/jacs.7b04829

[66] L. Lin , Q. Zhang , Y. Ni , L. Shang , X. Zhang , Z. Yan , Q. Zhao , J. Chen , Chem 2022, 8, 1822.

[67] M. Zhao , Y. Wang , Q. Ma , Y. Huang , X. Zhang , J. Ping , Z. Zhang , Q. Lu , Y. Yu , H. Xu , Y. Zhao , H. Zhang , Adv. Mater. 2015, 27, 7372. 26468970 10.1002/adma.201503648

[68] S. Li , B. Chen , Y. Wang , M. Y. Ye , P. A. van Aken , C. Cheng , A. Thomas , Nat. Mater. 2021, 20, 1240. 34059814 10.1038/s41563-021-01006-2

[69] F. Cao , M. Zhao , Y. Yu , B. Chen , Y. Huang , J. Yang , X. Cao , Q. Lu , X. Zhang , Z. Zhang , C. Tan , H. Zhang , J. Am. Chem. Soc. 2016, 138, 6924. 27197611 10.1021/jacs.6b02540

[70] R. Makiura , S. Motoyama , Y. Umemura , H. Yamanaka , O. Sakata , H. Kitagawa , Nat. Mater. 2010, 9, 565. 20512155 10.1038/nmat2769

[71] Y. Luo , M. Ahmad , A. Schug , M. Tsotsalas , Adv. Mater. 2019, 31, 1901744. 10.1002/adma.201901744 31106914

[72] N. Swain , B. Saravanakumar , M. Kundu , L. Schmidt‐Mende , A. Ramadoss , J. Mater. Chem. A 2021, 9, 25286.

[73] K. Yu , D.‐I. Won , W. I. Lee , W.‐S. Ahn , Korean J. Chem. Eng. 2021, 38, 653.

[74] M. Rimoldi , A. J. Howarth , M. R. DeStefano , L. Lin , S. Goswami , P. Li , J. T. Hupp , O. K. Farha , ACS Catal. 2017, 7, 997.

[75] Y. S. Wei , M. Zhang , R. Q. Zou , Q. Xu , Chem. Rev. 2020, 120, 12089. 32356657 10.1021/acs.chemrev.9b00757

[76] R. Matheu , E. Gutierrez‐Puebla , M. A. Monge , C. S. Diercks , J. Kang , M. S. Prevot , X. K. Pei , N. Hanikel , B. Zhang , P. D. Yang , O. M. Yaghi , J. Am. Chem. Soc. 2019, 141, 17081. 31613614 10.1021/jacs.9b09298

[77] E. X. Chen , M. Qiu , Y. F. Zhang , Y. S. Zhu , L. Y. Liu , Y. Y. Sun , X. Bu , J. Zhang , Q. Lin , Adv. Mater. 2018, 30, 1704388. 10.1002/adma.201704388 29178432

[78] W. Morris , B. Volosskiy , S. Demir , F. Gandara , P. L. McGrier , H. Furukawa , D. Cascio , J. F. Stoddart , O. M. Yaghi , Inorg. Chem. 2012, 51, 6443. 22676251 10.1021/ic300825s

[79] P. Deria , D. A. Gomez‐Gualdron , I. Hod , R. Q. Snurr , J. T. Hupp , O. K. Farha , J. Am. Chem. Soc. 2016, 138, 14449. 27768297 10.1021/jacs.6b09113

[80] D. Feng , Z. Y. Gu , Y. P. Chen , J. Park , Z. Wei , Y. Sun , M. Bosch , S. Yuan , H. C. Zhou , J. Am. Chem. Soc. 2014, 136, 17714. 25479454 10.1021/ja510525s

[81] D. Feng , W.‐C. Chung , Z. Wei , Z.‐Y. Gu , H.‐L. Jiang , Y.‐P. Chen , D. J. Darensbourg , H.‐C. Zhou , J. Am. Chem. Soc. 2013, 135, 17105. 24125517 10.1021/ja408084j

[82] H. L. Jiang , D. Feng , K. Wang , Z. Y. Gu , Z. Wei , Y. P. Chen , H. C. Zhou , J. Am. Chem. Soc. 2013, 135, 13934. 23984878 10.1021/ja406844r

[83] D. Feng , H. L. Jiang , Y. P. Chen , Z. Y. Gu , Z. Wei , H. C. Zhou , Inorg. Chem. 2013, 52, 12661. 24147847 10.1021/ic4018536

[84] Y. Bai , Y. B. Dou , L. H. Xie , W. Rutledge , J. R. Li , H. C. Zhou , Chem. Soc. Rev. 2016, 45, 2327. 26886869 10.1039/c5cs00837a

[85] K. Wang , X. L. Lv , D. Feng , J. Li , S. Chen , J. Sun , L. Song , Y. Xie , J. R. Li , H. C. Zhou , J. Am. Chem. Soc. 2016, 138, 914. 26717254 10.1021/jacs.5b10881

[86] X. L. Lv , K. Wang , B. Wang , J. Su , X. Zou , Y. Xie , J. R. Li , H. C. Zhou , J. Am. Chem. Soc. 2017, 139, 211. 27936748 10.1021/jacs.6b09463

[87] N. Huang , K. Wang , H. Drake , P. Cai , J. Pang , J. Li , S. Che , L. Huang , Q. Wang , H. C. Zhou , J. Am. Chem. Soc. 2018, 140, 6383. 29719956 10.1021/jacs.8b02710

[88] D. Y. Du , J. S. Qin , S. L. Li , Z. M. Su , Y. Q. Lan , Chem. Soc. Rev. 2014, 43, 4615. 24676127 10.1039/c3cs60404g

[89] M. L. Sun , Y. R. Wang , W. W. He , R. L. Zhong , Q. Z. Liu , S. Xu , J. M. Xu , X. L. Han , X. Ge , S. L. Li , Y. Q. Lan , A. M. Al‐Enizi , A. Nafady , S. Ma , Small 2021, 17, 2100762. 10.1002/smll.202100762 33817965

[90] D. Yang , B. Ni , X. Wang , Adv. Energy Mater. 2020, 10, 2001142.

[91] C. L. Hill , Chem. Rev. 1998, 98, 1.11851497

[92] Y. R. Wang , Q. Huang , C. T. He , Y. Chen , J. Liu , F. C. Shen , Y. Q. Lan , Nat. Commun. 2018, 9, 4466. 30367039 10.1038/s41467-018-06938-z PMC6203756

[93] A. P. Cote , A. I. Benin , N. W. Ockwig , M. O'Keeffe , A. J. Matzger , O. M. Yaghi , Science 2005, 310, 1166. 16293756 10.1126/science.1120411

[94] K. Wang , D. D. Qi , Y. L. Li , T. Y. Wang , H. B. Liu , J. Z. Jiang , Coord. Chem. Rev. 2019, 378, 188.

[95] M. Calik , F. Auras , L. M. Salonen , K. Bader , I. Grill , M. Handloser , D. D. Medina , M. Dogru , F. Loebermann , D. Trauner , A. Hartschuh , T. Bein , J. Am. Chem. Soc. 2014, 136, 17802. 25412210 10.1021/ja509551m PMC4706362

[96] X. Chen , M. Addicoat , E. Jin , L. Zhai , H. Xu , N. Huang , Z. Guo , L. Liu , S. Irle , D. Jiang , J. Am. Chem. Soc. 2015, 137, 3241. 25706112 10.1021/ja509602c

[97] A. P. Cote , H. M. El‐Kaderi , H. Furukawa , J. R. Hunt , O. M. Yaghi , J. Am. Chem. Soc. 2007, 129, 12914. 17918943 10.1021/ja0751781

[98] Z.‐J. Xia , H.‐C. Yang , Z. Chen , R. Z. Waldman , Y. Zhao , C. Zhang , S. N. Patel , S. B. Darling , Adv. Mater. Interfaces 2019, 6, 1900254.

[99] S. J. Lyle , P. J. Waller , O. M. Yaghi , Trends Chem. 2019, 1, 172.

[100] F. Chen , H. Zhu , N. Lv , Q. Li , T. Ma , L. Wang , M. Zhou , S. Cao , X. Luo , C. Cheng , Chemistry 2022, 28, 202104591. 10.1002/chem.202104591 35394659

[101] J. He , Z. Zhang , Y. Yang , F. Ren , J. Li , S. Zhu , F. Ma , R. Wu , Y. Lv , G. He , B. Guo , D. Chu , Nano‐Micro Lett. 2021, 13, 80. 10.1007/s40820-020-00585-0 PMC8187506 34138263

[102] Y. Deng , Y. Wang , Y. B. Chen , Z. Zhang , Chem. ‐Asian J. 2021, 16, 1851. 34002483 10.1002/asia.202100357

[103] E. L. Spitler , W. R. Dichtel , Nat. Chem. 2010, 2, 672. 20651731 10.1038/nchem.695

[104] X. A. Feng , L. Chen , Y. P. Dong , D. L. Jiang , Chem. Commun. 2011, 47, 1979. 10.1039/c0cc04386a 21221442

[105] S. Wan , F. Gandara , A. Asano , H. Furukawa , A. Saeki , S. K. Dey , L. Liao , M. W. Ambrogio , Y. Y. Botros , X. F. Duan , S. Seki , J. F. Stoddart , O. M. Yaghi , Chem. Mater. 2011, 23, 4094.

[106] Y. F. Zhu , D. Y. Zhu , Y. Chen , Q. Q. Yan , C. Y. Liu , K. X. Ling , Y. F. Liu , D. J. Lee , X. W. Wu , T. P. Senftle , R. Verduzco , Chem. Sci. 2021, 12, 16092. 35024131 10.1039/d1sc05379e PMC8672717

[107] Z. Meng , R. M. Stolz , K. A. Mirica , J. Am. Chem. Soc. 2019, 141, 11929. 31241936 10.1021/jacs.9b03441

[108] C. Singh , T. W. Kim , R. K. Yadav , J. O. Baeg , V. Gole , A. P. Singh , Appl. Surf. Sci. 2021, 544, 148938.

[109] X. Y. Pan , J. Q. Zhang , H. Zhou , R. H. Liu , D. J. Wu , R. Wang , L. P. Shen , L. Tao , J. Zhang , H. Wang , Nano‐Micro Lett. 2021, 13, 70. 10.1007/s40820-021-00596-5 PMC8187591 34138321

[110] B. Han , X. Ding , B. Q. Yu , H. Wu , W. Zhou , W. P. Liu , C. Y. Wei , B. T. Chen , D. D. Qi , H. L. Wang , K. Wang , Y. L. Chen , B. L. Chen , J. Z. Jiang , J. Am. Chem. Soc. 2021, 143, 7104. 33939427 10.1021/jacs.1c02145

[111] R. F. Chen , J. L. Shi , Y. Ma , G. Q. Lin , X. J. Lang , C. Wang , Angew. Chem., Int. Ed. 2019, 58, 6430. 10.1002/anie.201902543 30884054

[112] S. Kandambeth , D. B. Shinde , M. K. Panda , B. Lukose , T. Heine , R. Banerjee , Angew. Chem., Int. Ed. 2013, 52, 13052. 10.1002/anie.201306775 24127339

[113] A. Khaligh , Y. Sheidaei , D. Tuncel , ACS Appl. Energy Mater. 2021, 4, 3535.

[114] B. Nath , W. H. Li , J. H. Huang , G. E. Wang , Z. H. Fu , M. S. Yao , G. Xu , CrystEngComm 2016, 18, 4259.

[115] W. Ma , P. Yu , T. Ohsaka , L. Mao , Electrochem. Commun. 2015, 52, 53.

[116] S. Bhunia , K. A. Deo , A. K. Gaharwar , Adv. Funct. Mater. 2020, 30, 2002046. 10.1002/adfm.202003579 PMC7401402 32774203

[117] S. Lin , C. S. Diercks , Y.‐B. Zhang , N. Kornienko , E. M. Nichols , Y. Zhao , A. R. Paris , D. Kim , P. Yang , O. M. Yaghi , C. J. Chang , Science 2015, 349, 1208. 26292706 10.1126/science.aac8343

[118] C. S. Diercks , S. Lin , N. Komienko , E. A. Kapustin , E. M. Nichols , C. H. Zhu , Y. B. Zhao , C. J. Chang , O. M. Yaghi , J. Am. Chem. Soc. 2018, 140, 1116. 29284263 10.1021/jacs.7b11940

[119] Q.‐Y. Liu , J.‐F. Li , J.‐W. Wang , J. Inclusion Phenom. Macrocyclic Chem. 2019, 95, 1.

[120] G. Q. Lin , H. M. Ding , R. F. Chen , Z. K. Peng , B. S. Wang , C. Wang , J. Am. Chem. Soc. 2017, 139, 8705. 28595005 10.1021/jacs.7b04141

[121] S. Kumar , M. Y. Wani , C. T. Arranja , J. D. E. Silva , B. Avula , A. Sobral , J. Mater. Chem. A 2015, 3, 19615.

[122] W. Ji , T.‐X. Wang , X. Ding , S. Lei , B.‐H. Han , Coord. Chem. Rev. 2021, 439, 213875.

[123] W. R. Zheng , C. S. Tsang , L. Y. S. Lee , K. Y. Wong , Mater. Today Chem. 2019, 12, 34.

[124] T. Y. Wu , Z. Y. Ma , Y. Y. He , X. J. Wu , B. Tang , Z. Y. Yu , G. Wu , S. Chen , N. Z. Bao , Angew. Chem., Int. Ed. 2021, 60, 10366. 10.1002/anie.202101648 33594767

[125] H. Wang , H. Wang , Z. W. Wang , L. Tang , G. M. Zeng , P. Xu , M. Chen , T. Xiong , C. Y. Zhou , X. Y. Li , D. N. Huang , Y. Zhu , Z. X. Wang , J. W. Tang , Chem. Soc. Rev. 2020, 49, 4135. 32421139 10.1039/d0cs00278j

[126] C. Liu , H. Li , F. Liu , J. Chen , Z. Yu , Z. Yuan , C. Wang , H. Zheng , G. Henkelman , L. Wei , Y. Chen , J. Am. Chem. Soc. 2020, 142, 21861. 33332110 10.1021/jacs.0c10636

[127] C. Y. Lin , L. Zhang , Z. Zhao , Z. Xia , Adv. Mater. 2017, 29, 1606635. 10.1002/adma.201606635 28230916

[128] W. Q. Q. Zheng , R. Zhu , H. J. J. Wu , T. Ma , H. J. J. Zhou , M. Zhou , C. He , X. K. K. Liu , S. Li , C. Cheng , Angew. Chem., Int. Ed. 2022, 61, 202208667. 10.1002/anie.202208667 35876718

[129] Z. Zhang , L. Zhang , X. Wang , T. Wang , C. Cheng , X. Liu , ChemNanoMat 2021, 8, 202100345.

[130] M. Lu , J. Liu , Q. Li , M. Zhang , M. Liu , J. L. Wang , D. Q. Yuan , Y. Q. Lan , Angew. Chem., Int. Ed. 2019, 58, 12392. 10.1002/anie.201906890 31270914

[131] J. X. Wu , S. Z. Hou , X. D. Zhang , M. Xu , H. F. Yang , P. S. Cao , Z. Y. Gu , Chem. Sci. 2019, 10, 2199. 30881645 10.1039/c8sc04344b PMC6385528

[132] Z. H. Wu , J. H. Yang , W. J. Shao , M. H. Cheng , X. L. Luo , M. Zhou , S. Li , T. Ma , C. Cheng , C. S. Zhao , Adv. Fiber Mater. 2022, 4, 774.

[133] X. J. Cao , T. Z. Wang , L. F. Jiao , Adv. Fiber Mater. 2021, 3, 210.

[134] B. Zhang , Z. H. Wu , W. J. Shao , Y. Gao , W. W. Wang , T. Ma , L. Ma , S. Li , C. Cheng , C. S. Zhao , Angew. Chem., Int. Ed. 2022, 61, 202115331. 10.1002/anie.202115331 PMC9306610 34936185

[135] P. Peng , L. Shi , F. Huo , C. Mi , X. Wu , S. Zhang , Z. Xiang , Sci. Adv. 2019, 5, 2322. 10.1126/sciadv.aaw2322 PMC6677550 31414045

[136] I. Hod , M. D. Sampson , P. Deria , C. P. Kubiak , O. K. Farha , J. T. Hupp , ACS Catal. 2015, 5, 6302.

[137] D. F. Gao , R. M. Aran‐Ais , H. S. Jeon , B. R. Cuenya , Nat. Catal. 2019, 2, 198.

[138] H. P. Yang , X. D. Wang , Q. Hu , X. Y. Chai , X. Z. Ren , Q. L. Zhang , J. H. Liu , C. X. He , Small Methods 2020, 4, 1900826.

[139] J. D. Yi , R. K. Xie , Z. L. Xie , G. L. Chai , T. F. Liu , R. P. Chen , Y. B. Huang , R. Cao , Angew. Chem., Int. Ed. 2020, 59, 23641. 10.1002/anie.202010601 32926542

[140] N. Kornienko , Y. Zhao , C. S. Kley , C. Zhu , D. Kim , S. Lin , C. J. Chang , O. M. Yaghi , P. Yang , J. Am. Chem. Soc. 2015, 137, 14129. 26509213 10.1021/jacs.5b08212

[141] B. X. Dong , S. L. Qian , F. Y. Bu , Y. C. Wu , L. G. Feng , Y. L. Teng , W. L. Liu , Z. W. Li , ACS Appl. Energy Mater. 2018, 1, 4662.

[142] Z. F. Xin , J. J. Liu , X. J. Wang , K. J. Shen , Z. B. Yuan , Y. F. Chen , Y. Q. Lan , ACS Appl. Mater. Interfaces 2021, 13, 54949. 10.1021/acsami.1c15187 34766753

[143] H. X. Zhong , M. Ghorbani‐Asl , K. H. Ly , J. C. Zhang , J. Ge , M. C. Wang , Z. Q. Liao , D. Makarov , E. Zschech , E. Brunner , I. M. Weidinger , J. Zhang , A. V. Krasheninnikov , S. Kaskel , R. H. Dong , X. L. Feng , Nat. Commun. 2020, 11, 1409. 32179738 10.1038/s41467-020-15141-y PMC7075876

[144] L. Pan , S. Ott , F. Dionigi , P. Strasser , Curr. Opin. Electrochem. 2019, 18, 61.

[145] P. Strasser , Acc. Chem. Res. 2016, 49, 2658. 27797179 10.1021/acs.accounts.6b00346

[146] F. M. Sapountzi , J. M. Gracia , C. J. Weststrate , H. O. A. Fredriksson , J. W. Niemantsverdriet , Prog. Energy Combust. Sci. 2017, 58, 1.

[147] H. Zhong , L. A. Estudillo‐Wong , Y. Gao , Y. Feng , N. Alonso‐Vante , ACS Appl. Mater. Interfaces 2020, 12, 21605. 32309924 10.1021/acsami.0c02884

[148] J. H. Zagal , M. T. M. Koper , Angew. Chem., Int. Ed. 2016, 55, 14510. 10.1002/anie.201604311 27666439

[149] R. Bashyam , P. Zelenay , Nature 2006, 443, 63. 16957726 10.1038/nature05118

[150] Y. Li , W. Zhou , H. Wang , L. Xie , Y. Liang , F. Wei , J.‐C. Idrobo , S. J. Pennycook , H. Dai , Nat. Nanotechnol. 2012, 7, 394. 22635099 10.1038/nnano.2012.72

[151] Y. Jiao , Y. Zheng , M. Jaroniec , S. Z. Qiao , Chem. Soc. Rev. 2015, 44, 2060. 25672249 10.1039/c4cs00470a

[152] Q. Zhang , J. Guan , Adv. Funct. Mater. 2020, 30, 2000768.

[153] H.‐F. Wang , L. Chen , H. Pang , S. Kaskel , Q. Xu , Chem. Soc. Rev. 2020, 49, 1414. 32039429 10.1039/c9cs00906j

[154] K. Gong , F. Du , Z. Xia , M. Durstock , L. Dai , Science 2009, 323, 760. 19197058 10.1126/science.1168049

[155] S. Samanta , P. K. Das , S. Chatterjee , A. Dey , J. Porphyrins Phthalocyanines 2015, 19, 92.

[156] M. T. M. Koper , Chem. Sci. 2013, 4, 2710.

[157] M. T. M. Koper , J. Electroanal. Chem. 2011, 660, 254.

[158] L. Zhou , S. Y. Lu , S. Guo , SusMat 2021, 1, 194.

[159] S. S. Li , J. R. Sun , J. Q. Guan , Chin. J. Catal. 2021, 42, 511.

[160] D. Micheroni , G. X. Lan , W. B. Lin , J. Am. Chem. Soc. 2018, 140, 15591. 30392362 10.1021/jacs.8b09521

[161] D. G. Nocera , Inorg. Chem. 2009, 48, 10001. 19775081 10.1021/ic901328v PMC3332084

[162] M. D. Karkas , O. Verho , E. V. Johnston , B. Akermark , Chem. Rev. 2014, 114, 11863. 25354019 10.1021/cr400572f

[163] H. X. Jia , Y. C. Yao , J. T. Zhao , Y. Y. Gao , Z. L. Luo , P. W. Du , J. Mater. Chem. A 2018, 6, 1188.

[164] S. Karak , K. Dey , R. Banerjee , Adv. Mater. 2022, 34, 2202751. 10.1002/adma.202202751 35760553

[165] S. Zuo , Z. P. Wu , H. Zhang , X. W. Lou , Adv. Energy Mater. 2022, 12, 2103383.

[166] Z. Xu , Z. Liang , W. Guo , R. Zou , Coord. Chem. Rev. 2021, 436, 213824.

[167] W. Liao , S. Wang , H. Su , Y. Zhang , Nano Res. 2022, 10.1007/s12274-022-5048-1.

[168] K. Y. Zhu , X. F. Zhu , W. S. Yang , Angew. Chem., Int. Ed. 2019, 58, 1252.

[169] R. Zhu , W. Zheng , R. Yan , M. Wu , H. Zhou , C. He , X. Liu , C. Cheng , S. Li , C. Zhao , Adv. Funct. Mater. 2022, 32, 2207021.

[170] X. Ma , W. Luo , M. Yan , L. He , L. Mai , Nano Energy 2016, 24, 165.

